# A reduced proteomic signature in critically ill Covid-19 patients determined with plasma antibody micro-array and machine learning

**DOI:** 10.1186/s12014-024-09488-3

**Published:** 2024-05-17

**Authors:** Maitray A. Patel, Mark Daley, Logan R. Van Nynatten, Marat Slessarev, Gediminas Cepinskas, Douglas D. Fraser

**Affiliations:** 1 Epidemiology and Biostatistics, Western University, London, ON N6A 3K7 Canada; 2 Computer Science, Western University, London, ON N6A 3K7 Canada; 3 Medicine, Western University, London, ON N6A 3K7 Canada; 4 Lawson Health Research Institute, London, ON N6C 2R5 Canada; 5 Medical Biophysics, Western University, London, ON N6A 3K7 Canada; 6 Children’s Health Research Institute, London, ON N6C 4V3 Canada; 7 Pediatrics, Western University, London, ON N6A 3K7 Canada; 8 Clinical Neurological Sciences, Western University, London, ON N6A 3K7 Canada; 9 Physiology & Pharmacology, Western University, London, ON N6A 3K7 Canada; 10 London Health Sciences Centre, 800 Commissioners Road East, London, ON N6A 5W9 Canada

**Keywords:** COVID-19, Sepsis, Targeted proteomics, Machine learning, Organ System

## Abstract

**Background:**

COVID-19 is a complex, multi-system disease with varying severity and symptoms. Identifying changes in critically ill COVID-19 patients’ proteomes enables a better understanding of markers associated with susceptibility, symptoms, and treatment. We performed plasma antibody microarray and machine learning analyses to identify novel proteins of COVID-19.

**Methods:**

A case-control study comparing the concentration of 2000 plasma proteins in age- and sex-matched COVID-19 inpatients, non-COVID-19 sepsis controls, and healthy control subjects. Machine learning was used to identify a unique proteome signature in COVID-19 patients. Protein expression was correlated with clinically relevant variables and analyzed for temporal changes over hospitalization days 1, 3, 7, and 10. Expert-curated protein expression information was analyzed with Natural language processing (NLP) to determine organ- and cell-specific expression.

**Results:**

Machine learning identified a 28-protein model that accurately differentiated COVID-19 patients from ICU non-COVID-19 patients (accuracy = 0.89, AUC = 1.00, F1 = 0.89) and healthy controls (accuracy = 0.89, AUC = 1.00, F1 = 0.88). An optimal nine-protein model (PF4V1, NUCB1, CrkL, SerpinD1, Fen1, GATA-4, ProSAAS, PARK7, and NET1) maintained high classification ability. Specific proteins correlated with hemoglobin, coagulation factors, hypertension, and high-flow nasal cannula intervention (*P* < 0.01). Time-course analysis of the 28 leading proteins demonstrated no significant temporal changes within the COVID-19 cohort. NLP analysis identified multi-system expression of the key proteins, with the digestive and nervous systems being the leading systems.

**Conclusions:**

The plasma proteome of critically ill COVID-19 patients was distinguishable from that of non-COVID-19 sepsis controls and healthy control subjects. The leading 28 proteins and their subset of 9 proteins yielded accurate classification models and are expressed in multiple organ systems. The identified COVID-19 proteomic signature helps elucidate COVID-19 pathophysiology and may guide future COVID-19 treatment development.

**Supplementary Information:**

The online version contains supplementary material available at 10.1186/s12014-024-09488-3.

## Introduction

Severe acute respiratory syndrome coronavirus 2 (SARS-CoV-2) induces coronavirus disease 2019 (COVID-19), a pandemic disease affecting more than 750 million individuals with over 6.8 million deaths [1, 2]. COVID-19 vaccinations and alternative variants influence the incidence and severity of new COVID-19 cases [3–5]; consequently, an improved understanding of the disease is necessary to counteract possible vaccine breakthroughs [6, 7]. Individuals with COVID-19 present with heterogeneous symptoms and severity due to the complex, multi-system pathophysiological impact of the SARS-CoV-2 virus [8–11]. COVID-19 severity is also complicated by various demographic and clinical risk factors, including age, sex, and pre-existing comorbidities [12–15].

SARS-CoV-2 infection triggers an innate immune response characterized by elevations in plasma pro-inflammatory cytokines, proteases and related proteins [16–22]. Vascular injury and endothelial dysregulation are key components of COVID-19, often resulting in microvascular thrombosis [23–26]. A humoral immune response follows the innate reaction, with robust production of SARS-CoV-2-specific antibodies [27–29]. In critically ill patients, COVID-19 results in impaired immune cell homing and programmed cell death. Specifically, antigen presentation and B/T-cell function is reduced, neutrophils and M1-type macrophages are repurposed, endothelia and fibroblasts are disrupted, myeloid lines become reactive, and the extracellular matrix is altered [30]. Despite a wealth of knowledge on COVID-19 pathophysiology, a unique proteomic signature that (1) includes proteins expressed across multiple systems and (2) that can be used to identify novel connected pathways, remains elusive.

This study aims to identify proteins specific to critically ill COVID-19 patients relative to age- and sex-matched non-COVID-19 sepsis patients and healthy control subjects. Our specific objectives were: (1) to measure the concentrations of 2,000 plasma proteins with antibody microarrays from the three cohorts; (2) to determine the relative importance of the plasma proteins in identifying COVID-19 patients to develop classification models; (3) to correlate the leading proteins to clinically relevant variables; (4) to investigate expression changes in the leading proteins on hospitalization days 1, 3, 7, and 10; and (5) to determine the cell type and organ system expression patterns of the leading proteins.

## Methods

### Study participants, blood sampling, and Cohort Matching

We used the Sepsis 3.0 criteria, which does not require pathogen identification, to screen patients admitted to our intensive care unit (ICU) [31]. All COVID-19 participants were pre-vaccinated. Two SARS-CoV-2 viral genes (RdRP and E) were detected using a polymerase chain reaction to confirm or refute COVID-19 status [32]. Blood was drawn on ICU days 1, 3, 7, and 10 for COVID-19 patients and on ICU days 1 and 3 for non-COVID-19 patients, depending on their continued admission in the ICU. Blood was obtained via indwelling catheters, and if a venipuncture was required, research blood draws were coordinated with a clinically indicated blood draw. In keeping with accepted research phlebotomy protocols for adult patients, blood draws did not exceed maximal volumes [33]. Blood was centrifuged, the plasma isolated and aliquoted at 250 µL in cryovials, and frozen at − 80 °C. All samples remained frozen until use, and freeze/thaw cycles were avoided. The healthy control subjects were individuals without disease, acute illness, or prescription medications and whose samples were collected prior to the emergence of SARS-CoV-2 (Translational Research Centre, London, ON; Directed by Dr. D.D. Fraser) [34, 35]. Final participant groups were constructed by age- and sex-matching ICU COVID-19 patients with ICU non-COVID-19 sepsis controls and healthy control subjects, resulting in 15 participants per group.

### Patient demographics and Clinical Data

Baseline characteristics for COVID-19 and non-COVID-19 sepsis controls on ICU admission Day 1 were recorded, including age, sex, comorbidities, standard hospital laboratory measurements, PaO_2_ to FiO_2_ ratio, and chest radiograph findings. Also, the Multiple Organ Dysfunction Score (MODS) and Sequential Organ Failure Assessment Score (SOFA) were calculated [31, 36]. Clinical interventions received during the observation period were also recorded, including the use of antibiotics, antiviral agents, systemic corticosteroids, vasoactive medications, antiplatelet treatment, anticoagulation treatment, renal replacement therapy, high-flow oxygen therapy, and mechanical ventilation (both invasive and non-invasive). For healthy controls, only age and sex were available.

### Antibody microarray

The RayBio® L-Series Human Antibody Array 2000 kit (RayBiotech Life Inc., GA, USA) was used to measure 2,000 proteins in plasma obtained from age- and sex-matched COVID-19 and non-COVID-19 sepsis patients, as well as healthy control subjects. The kits detect a broad range of proteins, including, but not limited to, cytokines, growth factors, receptors, signalling proteins, metabolic enzymes, and epigenetic markers. Prior to sample analysis, the stored plasma cryotubes underwent visual inspection to ensure that they were sealed and intact. The plasma samples were then thawed, visually inspected, and centrifuged to remove low molecular weight amine derivaties and unwanted buffer (plasma samples were only used if free of obvious contamination, hemolysis, precipitate, and lipemia). A labelling reagent was applied to biotinylate the purified plasma. Before application of plasma, each lot of the antibody array slides was tested with a positive control to verify accuracy within a predefined range (e.g., CV%). A blocking buffer was then applied to the microarray glass slides, followed by the biotinylated plasma samples at a 20x dilution. A streptavidin-conjugated fluorescent dye (CY3-Equivelent) was applied, and protein expression was measured via laser fluorescence scanning. There were 4 arrays (“y”) that contained 493–507 protein targets (“X”; spots), for a total of 2000 protein targets. Of the 4 arrays, one was arbitrarily selected to be the “reference array”, to which all the other arrays were normalized. Background level subtraction was performed by measuring the local background around each spot and subtracting that from the measured spot fluorescence. Fluorescence levels were normalized as follows: X(Ny) = X(y) * P1/P(y); where: P1 = mean signal intensity of Positive Controls on reference array, P(y) = mean signal intensity of Positive Controls on Array “y”; X(y) = mean signal intensity for spot “X” on Array “y”; and X(Ny) = normalized signal intensity for spot “X” on Array “y”. The data used for our analysis was the normalized fluorescence intensity signals, which is an arbitrary unit refered to as RFI (relative fluorescence intensity).

### Conventional statistics

Patient baseline clinical characteristics (Day 1 of ICU admission) were reported as median (IQR) for continuous variables and frequency (%) for categorical variables. A Kruskal-Wallis comparison of the individual proteins was conducted between the healthy controls, ICU non-COVID-19 patients, and ICU COVID-19 patients on Day 1, followed by pairwise comparison with a post-hoc Dunn test that included a false discovery rate (Benjamini-Hochberg) correction. A paired comparison of protein expression on multiple days was conducted using the Wilcoxon Signed-Rank test with Bonferroni correction to assess changes during the ICU stay. Only multiple comparison-corrected *P*-values are reported, and those below 0.05 were considered statistically significant.

### Machine learning

The data was split into a feature selection dataset (70%) and a testing dataset (30%), stratified by subject groups (Supplemental Fig. 1). The feature selection was done on Day 1 data that combined healthy controls and ICU non-COVID-19 patients and compared them against ICU COVID-19 patients. The combined cohort ensures the selection of the most significant proteins relevant to both healthy controls and ICU non-COVID-19 patients, which may improve clinical translation. The Boruta feature selection algorithm, based on Random Forest classifiers, was used to identify the most important proteins [37]. It individually compares each protein to randomly arranged versions of the data to determine if the protein is better at classifying than chance. The results from the Boruta feature reduction identified the most relevant proteins for classifying COVID-19 (a “reduced protein signature”). To assess the classification ability, two separate Random Forest classifiers were created to assess healthy controls versus ICU COVID-19 patients and ICU non-COVID-19 patients versus ICU COVID-19 patients.

Steps were undertaken to conduct a conservative analysis that mitigates small sample sizes and overfitting concerns. The Boruta algorithm was run on the feature reduction dataset to determine the most relevant features. The testing dataset was modified to contain only the identified relevant features. The reduced testing dataset was then used for the classification of COVID-19 with a Random Forest classifier. To reduce overfitting and maintain a conservative model, three-fold cross-validation was used with a Random Forest of 10 trees and a maximum depth of 3 [38]. The accuracy, receiver operating characteristic (ROC) curve area-under-curve (AUC), and the F1 score are reported. A high F1 score indicated that precision and recall are high.

As a Random Forest is a set of decision trees, we were able to interrogate this collection of trees to identify the features that have the highest predictive value (viz., those features that frequently appear near the top of the decision tree). Based on this characteristic, recursive feature elimination (RFE) was used to prepare an optimal model. RFE started with the reduced training dataset, fitted a Random Forest classifier, dropped the least important feature, and repeated the process until only ten features remained. Due to the randomness of the algorithm and Random Forest models, 10,000 runs of RFE were conducted. Those features in the top 10 for more than a specified threshold of the 10,000 runs were determined to be the optimal features. An optimal testing dataset containing only these optimal features was generated from the reduced testing dataset. The same classification process for the reduced testing dataset was used on the optimal testing dataset.

The proteomic data was visualized with a nonlinear dimensionality reduction on the reduced and optimal datasets using the t-distributed stochastic nearest neighbour embedding (t-SNE) algorithm. A t-SNE assumes that the ‘optimal’ representation of the data lies on a manifold with complex geometry, but in a low dimension, embedded in the full-dimensional space of the raw data [39]. Seperate t-SNE plots were constructed with all participants on the complete, reduced, and optimal datasets for visual comparison of clustering patterns. A pairwise comparison, using cosine similarity, was conducted to determine the similarity between subjects across the selected proteins and time points [40]. As such, subjects similar across their selected proteomic profile have a score closer to 1, while dissimilar subjects have a score closer to 0. The analysis was done with data Min-Max scaled between 0 and 1, and the cosine similarities were visualized using a heatmap.

The indivudal protein performance for distinguishing ICU COVID-19 patients from healthy controls and ICU non-COVID-19 patients was compared using a bootstrap Logistic Regression approach with 1000 repetitions. The participants were sampled with replacement, and three-fold cross-validation was used. The mean ROC AUC, sensitivity, specificity, and the F1 score are reported. The machine learning analysis was conducted using Python version 3.10.11 and Scikit-Learn version 1.2.2 [41].

### Natural Language Processing

In order to identify physiological domains of interest in COVID-19 patients, exploratory expression analysis was conducted with Natural Language Processing (NLP). Expertly curated mRNA/protein expression information was parsed from the Uniprot Knowledgebase as unstructured text with UniProt’s REST API [42]. An NLP named-entity recognition (NER) pipeline was configured with the MIMIC package for preprocessing, negation detection, and the pretrained Stanza BioNLP13CG Biomedical model (Python v. 3.10.11; spaCy v. 3.3.1; spaCy-Stanza v. 1.0.2; negspaCy v. 1.0.3) [43–45]. The negation detection was done using the NegEx-based negspaCy implementation with a modified English clinical term set to filter negative expression terms. Although the BioNLP13CG biomedical model was based on Cancer Genetics and publicly available PubMed abstracts, compared to the other Stanza models, it provided the most granular entity classification, including anatomical system, organ, tissue, multi-level tissue, and cell type entities. The detected organ and cell type entities were manually classified into keyword-based groups separately. The manual expression curation process relies on existing literature and is not easily structured into specific organ systems. The organ, tissue, multi-tissue, and anatomical system entity types were combined and manually sorted into organ systems to include the maximum expression information in the analysis. The frequency of the keyword-based categories with respect to the relevant proteins was determined to identify physiological patterns of expression.

## Results

A total of three age- and sex-matched groups were included, consisting of COVID-19 patients (median years old = 60; IQR = 12; *n* = 15), non-COVID-19 sepsis patients (median years old = 57; IQR = 11; *n* = 15), and healthy control subjects (median years old = 56; IQR = 10; *n* = 15). There were no significant differences in age (Kruskal-Wallis H-test, *P* = 0.87) and sex (Chi-Square, *P* = 1.000) between the three cohorts. Baseline demographic characteristics, comorbidities, laboratory measurements, interventions, and chest x-ray findings of COVID-19 and non-COVID-19 sepsis controls are reported in Table 1. The two cohorts were generally similar in terms of their demographics, comorbidities, and interventions, except that COVID-19 patients had longer intubation periods and greater ICU days. While all ICU patients met the Sepsis 3.0 presentation criteria, only 40% of the non-COVID-19 ICU patients had pathogen identified. The COVID-19 patients were more likely to have bilateral pneumonia, lower white blood cell and lymphocyte counts, higher INR and PTT, and a lower PaO_2_/FiO_2_ ratio.

**Table 1:** Demographics and Clinical Variables of non-COVID-19 and COVID-19 ICU patients

Variable	Non-COVID-19 ICU (*n* = 15)	COVID-19 ICU (*n* = 15)	*P* Value
Age, median (IQR)	57.0 (52.0–63.0)	60.0 (53.0–65.0)	0.739
Male, no. (%)	7 (46.7)	7 (46.7)	1.000
Height (cm), median (IQR)	164.0 (159.1-172.5)	170.0 (163.5–173.0)	0.329
Weight (kg), median (IQR)	77.0 (64.6–97.8)	92.0 (81.6-107.5)	**0.044**
BMI, median (IQR)	28.4 (23.2–33.6)	30.7 (28.2–38.6)	0.135
SOFA, median (IQR)	7.0 (5.0–9.0)	5.0 (2.5–9.5)	0.318
MODS, median (IQR)	5.0 (3.5-8.0)	4.0 (3.5-6.0)	0.367
Sepsis Presentation, no. (%)	15 (100.0)	15 (100.0)	1.000
Pathogen Identified, no. (%)	6 (40.0)	15 (100.0)	**< 0.001**
**Comorbidities, no. (%)**			
Diabetes	6 (40.0)	5 (33.3)	1.000
Hypertension	10 (66.7)	7 (46.7)	0.462
Coronary Artery/Heart Disease	2 (13.3)	2 (13.3)	1.000
Chronic Heart Failure	2 (13.3)	0 (0.0)	0.483
Chronic Kidney Disease	1 (6.7)	2 (13.3)	1.000
Cancer	1 (6.7)	2 (13.3)	1.000
COPD	3 (20.0)	1 (6.7)	0.598
**Pulmonary pathology, no. (%)**			
Unilateral Pneumonia	8 (53.3)	1 (6.7)	**0.014**
Bilateral Pneumonia	1 (6.7)	14 (93.3)	**< 0.001**
Bilateral Opacities	1 (6.7)	--	--
Interstitial Infiltrate	2 (13.3)	--	--
**Laboratories, median (IQR)**			
Hemoglobin	124.0 (104.5-138.5)	121.0 (107.0-131.0)	0.547
White Blood Cell count	16.4 (12.0-21.2)	8.7 (7.0-16.2)	**0.031**
Neutrophils	12.7 (9.9–15.8)	7.7 (5.7–13.3)	0.055
Lymphocytes	1.4 (0.8–1.8)	0.8 (0.6-1.0)	**0.030**
Platelets	212.0 (173.0-262.0)	209.0 (163.5-301.5)	0.917
Creatinine	79.0 (53.5–98.5)	82.0 (63.0-190.0)	0.340
International Normalized Ratio	1.0 (1.0-1.1)	1.2 (1.2–1.3)	**0.006**
Lactate	1.5 (1.0-3.3)	1.7 (1.1–1.9)	0.803
Partial thromboplastin time (PTT)	23.0 (21.5–24.5)	28.0 (25.5–31.0)	**< 0.001**
PaO_2_/FiO_2_ Ratio	172.0 (137.8-290.8)	120.0 (69.5–153.0)	**0.026**
**Intervention, no. (%)**			
Renal Replacement Therapy	1 (6.7)	3 (20.0)	0.598
High-Flow Nasal Cannula	4 (26.7)	9 (60.0)	0.139
Non-Invasive Mechanical Ventilation	4 (26.7)	6 (40.0)	0.700
Invasive Mechanical Ventilation	14 (93.3)	11 (73.3)	0.330
Days Intubated, median (IQR)	4.0 (2.5-5.0)	14.0 (2.5–18.0)	**0.046**
Steroids	7 (46.7)	4 (26.7)	0.450
Vasoactive Medications	10 (66.7)	12 (80.0)	0.682
Antibiotics	15 (100.0)	15 (100.0)	1.000
Anti-virals	2 (13.3)	3 (20.0)	1.000
Antiplatelet	7 (46.7)	5 (33.3)	0.710
Anticoagulation	15 (100.0)	14 (93.3)	1.000
**Outcome**			
Death, no. (%)	2 (13.3)	7 (46.7)	0.109
ICU Days, median (IQR)	5.0 (4.5-6.0)	17.0 (11.0-24.5)	**< 0.001**

Note: *P* Value calculated with Mann-Whitney U test for continuous variables or Fisher Exact Test for binary variables

**Fig. 1:**
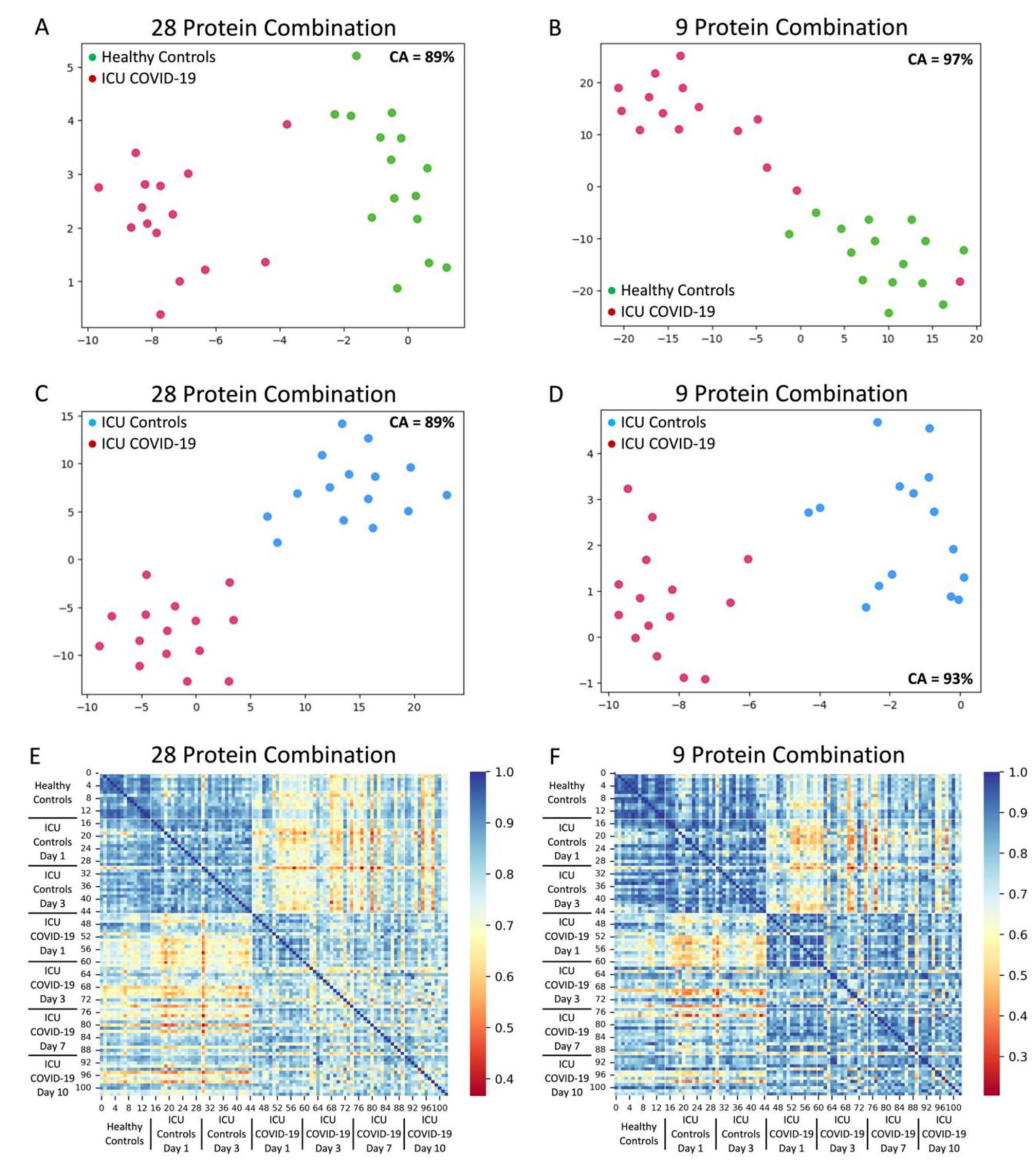
Identification of important plasma proteins in ICU COVID-19 patients. (**A**) Healthy controls compared to ICU COVID-19 Day 1 measurements plotted in two dimensions, following t-SNE dimensionality reduction of all 28 important proteins determined by Boruta feature reduction. The plot shows cluster separation of ICU COVID-19 patients from healthy control subjects, with one possible outlier. (**B**) Healthy controls compared to ICU COVID-19 Day 1 measurements plotted in two dimensions, following t-SNE dimensionality reduction of the top 9 important proteins determined by Recursive Feature Selection with a 50% threshold. The plot shows the cluster separation of ICU COVID-19 patients from healthy control subjects with one outlier. (**C**) ICU non-COVID-19 patients compared to ICU COVID-19 Day 1 measurements plotted in two dimensions, following t-SNE dimensionality reduction of all 28 important proteins determined by Boruta feature reduction. The plot shows the cluster separation of ICU COVID-19 patients from ICU non-COVID-19 subjects. (**D**) ICU non-COVID-19 patients compared to ICU COVID-19 Day 1 measurements plotted in two dimensions, following t-SNE dimensionality reduction of the top 9 important proteins determined by Recursive Feature Selection with a 50% threshold. The plot shows the cluster separation of ICU COVID-19 patients from ICU non-COVID-19 subjects. (**E**) A heatmap demonstrated the pairwise cosine similarity between cohorts’ protein profiles for the important 28 proteins across all timepoints. A greater cosine similarity measure between subjects indicates similar protein profiles, while a smaller measure indicates large differences between profiles. The protein profile of ICU COVID-19 patients is distinctively different from that of ICU non-COVID-19 and healthy control participants. (**F**) A heatmap demonstrated the pairwise cosine similarity between cohorts’ protein profiles with only the top 9 proteins across all timepoints. A greater cosine similarity measure between subjects indicates similar protein profiles, while a smaller measure indicates large differences between profiles. The protein profile of ICU COVID-19 patients is distinctively different from that of ICU non-COVID-19 and healthy control participants, with more homogeneity within each group

The expression levels of 2,000 proteins (1,968 unique proteins) were measured (Supplemental Fig. 2), and the cohorts plotted with t-SNE (all 2,000 proteins; Supplemental Fig. 3). Using Boruta feature selection machine learning, the leading 28 proteins were identified in comparing ICU COVID-19 patients to ICU non-COVID-19 patients (ICU day 1 for both) and healthy control subjects, and their relative importance is provided in Table 2. The leading 28 protein model had high classification ability when comparing ICU COVID-19 patients to ICU non-COVID-19 patients (accuracy = 0.89, AUC = 1.00, F1 = 0.89) as well as when comparing ICU COVID-19 patients to healthy control subjects (accuracy = 0.89, AUC = 1.00, F1 = 0.88). Individually, each of the 28 proteins was significantly different in COVID-19 patients compared to non-COVID-19 patients (FDR adjusted *P* < 0.05). When compared to healthy controls, 4 proteins out of the 28 (Galanin, ProSAAS, VimentinB, and NET1) were not significantly different from COVID-19 patients. Of the 28 proteins, only four had overall elevated levels in the COVID-19 patients (Fyn, Fen1, Azurocidin, and NET1; Supplemental Fig. 4). The individual classification abilities varied between the 28 proteins and is provided in Supplemental Tables 1 and Supplemental Table 2. Visualizing the 28 protein classification ability on Day 1 using t-SNE plots demonstrated a distinct COVID-19 patient cluster separation (one outlier) from healthy controls (Fig. 1A) as well as non-COVID-19 patients (Fig. 1C). The functions of the 28 proteins are described in Supplemental Table 3.

**Table 2:** Relative Fluorescence Intensity Cohort Comparison and Feature Importance of the 28 Proteins

Protein	Healthy Controls Subjects	non-COVID-19 ICU patients	COVID-19 ICU patients	*P* Value				Feature Importance %
				Kruskal-Wallis	Healthy vs. COVID	Non-COVID vs. COVID	Healthy vs. Non-COVID	
Nucleobindin1/NUCB1	2139.6 (2035.6-2526.9)	2767.9 (2349.5-3960.4)	1715.6 (1500.7-1977.1)	< 0.001	0.003	< 0.001	0.042	8.86
Fibronectin	45978.1 (41953.6-50954.6)	48802.8 (38226.8-60780.7)	29829.7 (25145.5-32101.9)	< 0.001	< 0.001	< 0.001	0.813	8.61
SerpinB5	31377.6 (22662.9-34870.9)	36270.3 (28011.4-42295.1)	18502.8 (14779.7-21861.6)	< 0.001	0.004	< 0.001	0.148	8.42
HSPA8	5332.2 (4701.7-6263.1)	5589.2 (4712.2-6968.6)	2951.1 (2420.5-4056.6)	< 0.001	< 0.001	< 0.001	0.597	8.12
ERRa	3357.6 (3191.4-3812.2)	3900.5 (3328.8-4948.5)	1301.7 (1107.9-1780.1)	< 0.001	< 0.001	< 0.001	0.541	7.91
SerpinA12	39283.3 (29516.8-42026.8)	55427.7 (45914.9-65692.0)	21207.7 (17283.3-26846.4)	< 0.001	0.010	< 0.001	0.036	7.84
Fyn	2347.3 (2083.6-2509.2)	1734.6 (1677.4-2289.4)	3838.5 (3208.9-4700.4)	< 0.001	< 0.001	< 0.001	0.331	7.41
GATA-4	1334.7 (1135.0-1454.9)	1070.0 (877.2-1186.5)	606.8 (485.2-660.6)	< 0.001	< 0.001	0.004	0.036	7.06
MammaglobinA	4569.8 (4269.2-5600.4)	4873.9 (3900.4-5373.9)	2944.7 (2434.6-3529.7)	< 0.001	< 0.001	< 0.001	0.559	4.54
SerpinD1	2622.1 (2282.0-2897.9)	2533.9 (1626.6–3197.0)	766.5 (623.2-1239.1)	< 0.001	< 0.001	< 0.001	0.627	4.21
Presenilin2	1007.6 (904.5-1145.2)	968.2 (884.8-1061.5)	629.2 (571.3-727.1)	< 0.001	< 0.001	0.003	0.352	2.99
SerpinA4	46385.1 (39766.8-48938.7)	43038.2 (36277.4-51919.7)	19742.5 (18351.1-24913.0)	< 0.001	< 0.001	< 0.001	0.889	2.87
PARK7	1008.3 (938.2–1224.0)	809.6 (627.4-1145.1)	390.1 (235.5-486.8)	< 0.001	< 0.001	0.002	0.211	2.55
IGFBP-5	1327.5 (1216.5-1438.3)	1437.6 (1257.0-1797.8)	808.3 (681.8-977.9)	< 0.001	0.011	< 0.001	0.211	2.48
HPR	4088.8 (3254.0-5490.0)	4883.1 (4162.4-6666.2)	2559.9 (1905.0-2956.7)	< 0.001	< 0.001	< 0.001	0.266	2.07
EphB4	2039.2 (1753.0-2486.6)	2281.4 (1431.5-2747.9)	781.8 (661.0-1212.6)	< 0.001	< 0.001	< 0.001	0.687	1.84
Fen1	1959.4 (1586.4-2183.9)	1701.8 (1165.3-2907.6)	3452.5 (3196.2-3699.9)	< 0.001	< 0.001	< 0.001	0.857	1.82
SHANK1	2106.9 (1993.2–2526.0)	3637.3 (2935.6-4592.3)	1646.0 (1420.8–1958.0)	< 0.001	0.024	< 0.001	0.013	1.46
CrkL	10605.7 (6730.2-15438.9)	13671.4 (8277.4-23294.7)	3775.9 (3093.4-7097.2)	< 0.001	0.007	< 0.001	0.206	1.43
Azurocidin	736.7 (649.8-914.3)	786.3 (458.5-980.9)	1249.0 (1041.2-1458.9)	< 0.001	< 0.001	< 0.001	0.846	1.33
PCMT1	2732.7 (1904.8-2965.8)	2860.9 (2242.7-4468.6)	1699.3 (1395.0-1943.7)	< 0.001	0.005	< 0.001	0.144	1.22
SerpinA1	56368.0 (49094.9-65906.2)	92996.3 (72774.3-110845.8)	38358.2 (35020.4-47527.1)	< 0.001	0.029	< 0.001	0.004	1.18
Proteasome26SS5	2571.5 (2123.1-3126.2)	2930.7 (2679.6-3663.4)	1827.8 (1709.8-1996.8)	< 0.001	0.011	< 0.001	0.148	0.93
PF4V1	2995.3 (2579.3–3466.0)	4117.6 (3632.9-4974.6)	2172.4 (2067.1–2461.0)	< 0.001	0.011	< 0.001	0.037	0.81
Galanin	61227.5 (52182.3-70312.9)	96782.7 (88396.2-103526.7)	46296.2 (34583.3-55448.2)	< 0.001	0.141	< 0.001	< 0.001	0.75
ProSAAS	79695.5 (55365.2-90619.9)	124482.4 (107774.4-140386.0)	43001.7 (36282.0-79197.1)	< 0.001	0.196	< 0.001	0.002	0.59
VimentinB	1348.7 (1236.9–1570.0)	2424.8 (1881.2-3601.1)	1082.3 (757.1-1418.7)	< 0.001	0.073	< 0.001	0.005	0.37
NET1	6309.6 (5222.1–7691.0)	3231.5 (2128.9-4140.6)	7573.7 (6916.4-8694.5)	< 0.001	0.130	< 0.001	0.002	0.33

Note: Relative Fluorescence Intensity is an arbitrary unit. Three groups compared with Kruskal-Wallis and pairwise groups compared with a Post-hoc Dunn Test. All tests corrected for using False Discovery Rate (Benjamini-Hochberg). Feature Importance represents the combined group (healthy control and non-COVID-19 ICU patients) vs. ICU COVID-19 patients

Recursive feature elimination was used to determine a set of optimal proteins. Those proteins in the top 10 for at least 5,000 of the 10,000 RFE repetitions (50%) were selected as the optimal protein model. Nine of the 28 proteins were optimal: PF4V1, NUCB1, CrkL, SerpinD1, Fen1, GATA-4, ProSAAS, PARK7, and NET1 (Supplemental Fig. 5). The optimal set of proteins maintained a high classification ability between COVID-19 patients on Day 1 and healthy controls (accuracy = 0.97, ROC = 0.97, F1 = 0.96) as well as between COVID-19 and non-COVID-19 on Day 1 (accuracy = 0.93, ROC = 1.00, F1 = 0.92). All proteins were significantly different in COVID-19 patients from non-COVID-19 patients, while ProSAAS and NET1 were not significantly different between COVID-19 and healthy controls (FDR-adjusted *P* < 0.05). Of the 9 proteins, only Fen1 and NET1 were elevated in COVID-19 patients. Visually, the t-SNE plots based on the nine optimal proteins illustrate a separation between the COVID-19 patients and healthy controls, with two outliers (Fig. 1B) as well as a distinct separation between COVID-19 patients and ICU non-COVID-19 patients (Fig. 1D).

Pairwise cosine similarity between all subjects and available time points was calculated to compare the cohorts in terms of their reduced and optimal protein profiles, presented in Fig. 1E and F, respectively. The healthy control subjects have the most homogenous protein profiles in both the 28 and 9 protein models. The non-COVID-19 sepsis controls were relatively homogenous across ICU Days 1 and 3, with observable differences from healthy control subjects. The COVID-19 patients are distinct at all time points from the other cohorts. Compared to the 28 protein profile, the COVID-19 patients are more homogenous across time points with the 9 protein profile. The expression of the leading proteins in COVID-19 patients on ICU Days 3, 7, and 10 were compared to their ICU Day 1 expression and demonstrated no significant differences over time (*P* > 0.05; data not shown).

**Fig. 2:**
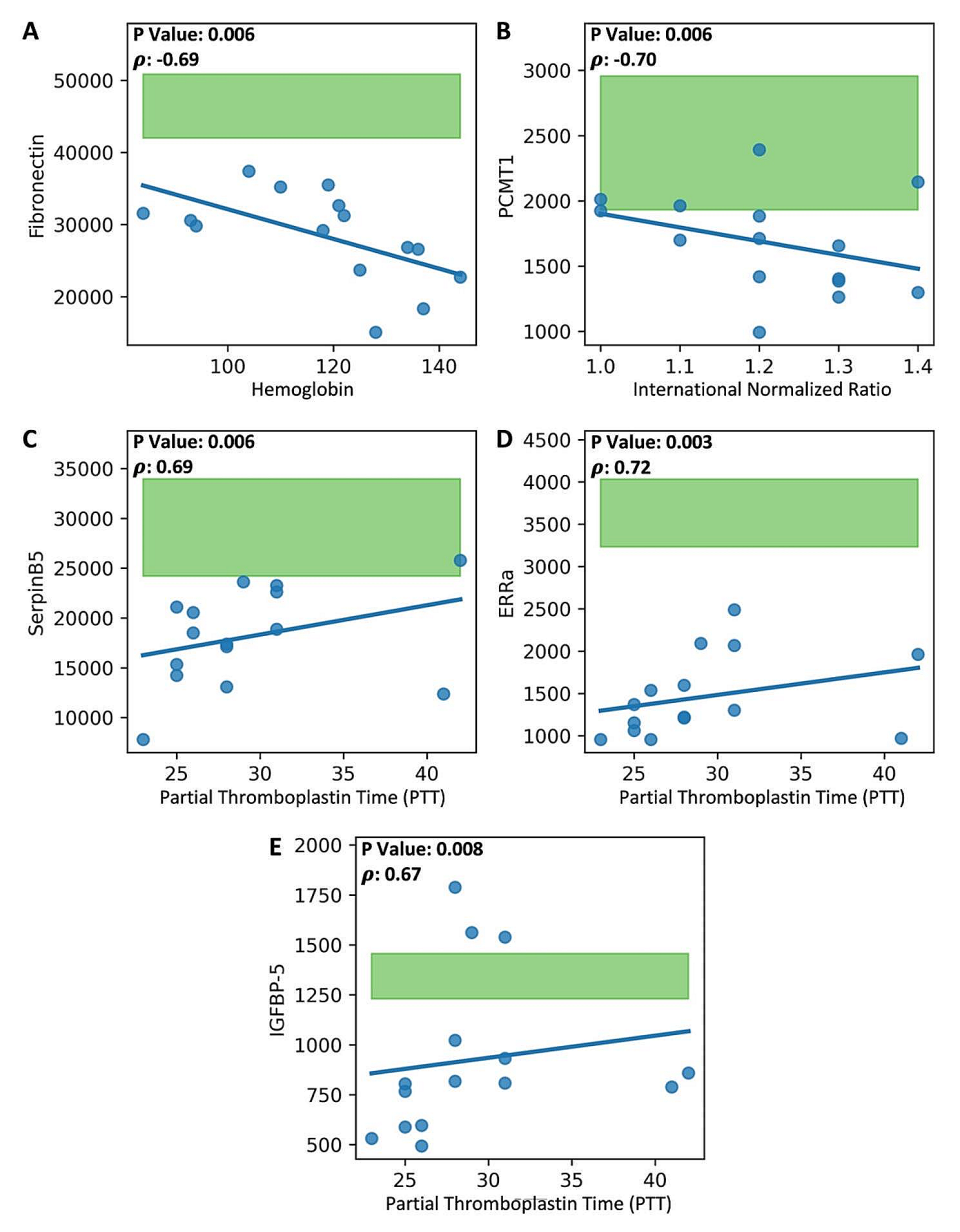
Correlations between important 28 proteins and continuous clinical variables in ICU COVID-19 patients. Blue points are ICU COVID-19 measurements; the green-filled area represents the 5th percentile to 95th percentile protein expression range of healthy control subjects. Only significant correlations (*p* < 0.01) are shown. The correlation coefficient and *P* Value per comparison are shown. (**A-B**) Plots demonstrating decreased protein expression in COVID-19 compared to healthy controls for Fibronectin and PCMT1. Fibronectin is significantly negatively correlated with hemoglobin (*p* = 0.006), and PCMT1 is significantly negatively correlated with the International Normalized Ratio (*p* = 0.006). (**C-E**) Plots demonstrating reduced protein expression in COVID-19 compared to healthy controls for SerpinB5, EERa, and IGFBP-5. Each protein, SerpinB5, EERa, and IGFBP-5, is significantly positively correlated with Partial Thromboplastin Time (*p* = 0.006, *p* = 0.003, *p* = 0.008, respectively)

**Fig. 3:**
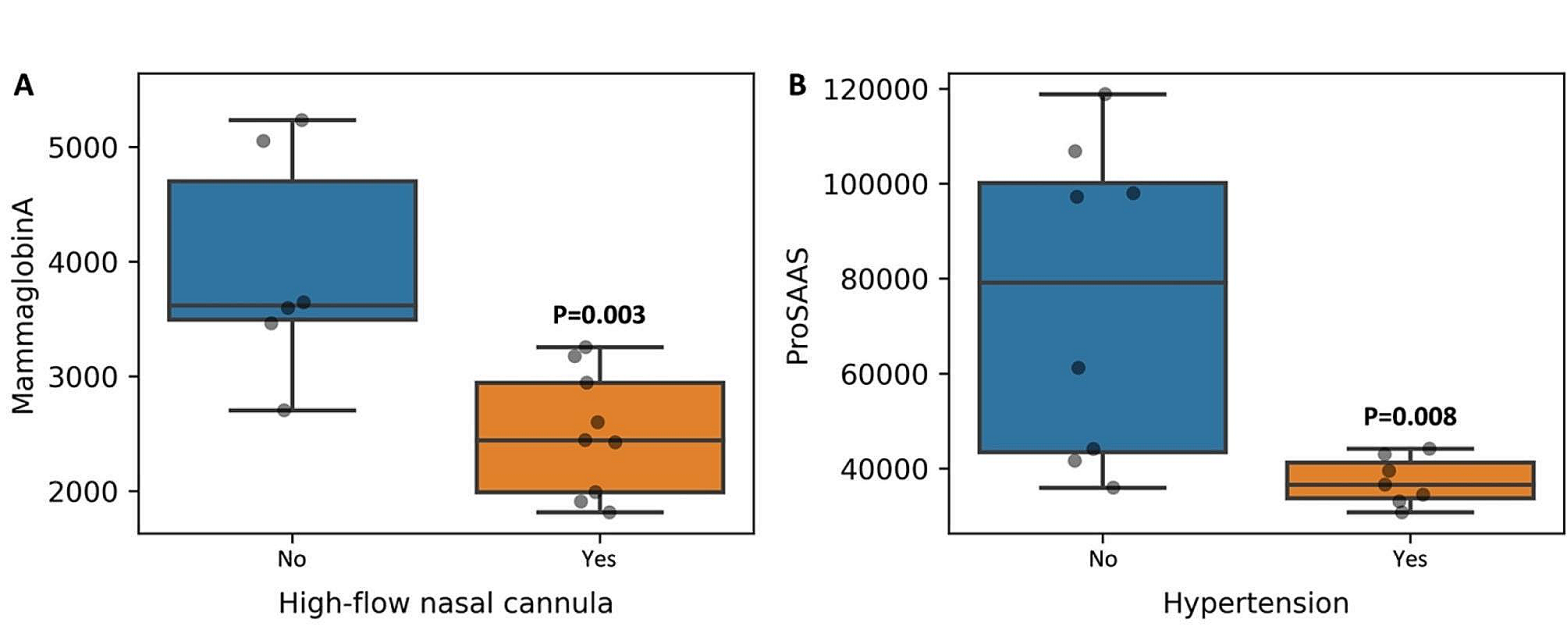
Differences in the important 28 proteins relative to binary clinical variables in ICU COVID-19 patients. (**A**) A box plot demonstrating that MammaglobinA is significantly elevated in those that didn’t receive high-flow nasal cannula (*p* = 0.003). (**B**) A box plot demonstrating that ProSAAS is significantly lower in those who had hypertension (*p* = 0.009)

The relevant leading 28 protein measurements of the COVID-19 patients were compared to their clinical variables. A total of seven significant associations (*P* < 0.01) were identified and are presented in Figs. 2 and 3. Fibronectin levels in all COVID-19 patients were below healthy control subjects and demonstrated a negative correlation with hemoglobin (Fig. 2A). Most COVID-19 patients’ PCTM1 measurements were below healthy control subjects and negatively correlated with INR (Fig. 2B). SerpinB5, ERRa, and IGFBP-5 in COVID-19 patients were all positively correlated with PTT, and most patients had measurement levels below healthy control subjects (Fig. 2C-E). MammaglobinA was lower in COVID-19 patients who received high-flow nasal cannula intervention (Fig. 3A). ProSAAS was lower in patients with hypertension comorbidity (Fig. 3B).

**Fig. 4:**
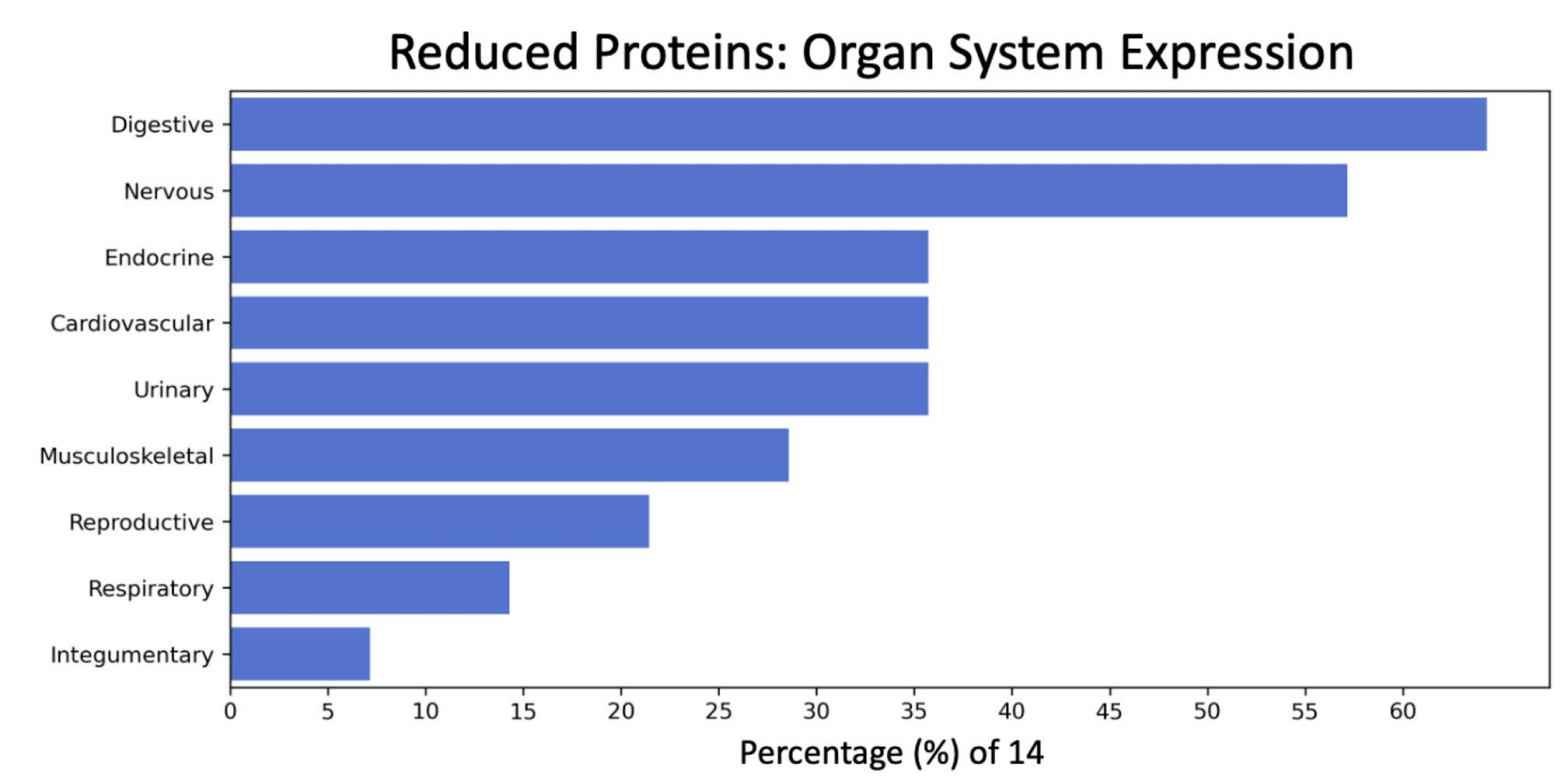
Frequency of protein expression in major organs/body systems. A bar plot demonstrates the percentage of proteins that are expressed in specific major organs and body systems as determined by Natural Language Processing (NLP). There were 14 proteins, out of the 28 proteins (50%), with UniProt organ system expression information. The organ system classification combines NLP-identified organs, tissue, multi-level tissue, and anatomical system entities. The lymphatic system did not have any associated proteins and was not shown for visualization clarity

Named-entity recognition was conducted on the tissue expression information provided by the UniProt Knowledgebase. Out of the 28 leading proteins, 14 (50%) had organ expression information (Supplemental Table 4), and 8 (29%) had cell type expression information (Supplemental Table 5). The percentage of the 14 proteins expressed in specific organ systems, led by the digestive and nervous systems, is shown in Fig. 4. The percentage of the eight proteins expressed in specific cell types is shown in Supplemental Fig. 6.

## Discussion

In this study, we measured the expression of 2,000 plasma proteins with antibody micro-array technology from age- and sex-matched COVID-19 patients, non-COVID-19 sepsis controls, and healthy control subjects. Using machine learning-based protein subset identification, we identified a 28-protein model that accurately differentiated COVID-19 patients from their comparison cohorts. Furthermore, we determined an optimal 9-protein subset model that maintained high classification ability. Some identified proteins were associated with clinical and demographic characteristics in the COVID-19 patients. NLP of expert-curated expression information identified multi-system expression of the leading proteins. This study has identified a reduced protein signature for COVID-19 patients that contributes to COVID-19 pathophysiology characterization and may inform the development of therapeutic interventions upon further investigation.

Our critically ill COVID-19 cohort was similar to other reported cohorts, with only minor differences [8, 46–49]. For example, the mortality rate in our COVID-19 patients was higher than reported by other studies and may suggest a greater illness burden in our patients [8, 46, 50]. The platelet count in our COVID-19 patients was lower than reported in the literature [51–53], perhaps reflecting greater microvascular injury and overall microclot risk [23]. Similarly, the PaO_2_/FiO_2_ ratio was also lower in our COVID-19 patients [8, 53], indicating higher levels of acute lung injury. Although COVID-19 lymphocyte counts, INR, and bilateral pulmonary complications were significantly different than in non-COVID-19 sepsis controls, they were similar to those in COVID-19 patients reported in the literature [49, 52, 53].

A unique 28-protein signature that differentiated COVID-19 patients from non-COVID-19 sepsis controls and healthy control subjects was determined. Each of the identified proteins was individually different in the COVID-19 cohort from the non-COVID-19 cohort, as well as 24 proteins were different in the healthy control subjects. Many proteins had high individual distinguishing power, further positioning them as possible disease biomarkers. Those proteins with weaker individual performance may be beneficial in a combination or secondary role. Time-based analysis and inspection of the pairwise subject comparison demonstrated no changes in COVID-19 protein expression over multiple ICU days and interventions, suggesting that the reduced protein signature is robust, reproducible, and remains highly predictive of COVID-19 disease status over 10 hospitalization days. In addition, an optimal model consisting of 9 proteins (PF4V1, NUCB1, CrkL, SerpinD1, Fen1, GATA-4, ProSAAS, PARK7, and NET1) maintained the high classification ability found in the superset 28-protein model. The pairwise comparison analysis suggests that the nine-protein model may be more consistent across multiple days than the 28-protein model.

Correlation analysis comparing the expression of the 28-protein in COVID-19 patients with their respective clinical characteristics identified seven associations. Interestingly, five proteins correlated with measures of blood clotting, including the INR and PTT. The COVID-19 patients had significantly higher INR and PTT measurements compared to non-COVID-19 sepsis controls; however, the measurements were within the normal clinical range. Almost all patients across the two ICU cohorts had anticoagulation interventions. PCMT1 was negatively correlated with INR in COVID-19 patients but not linked to thrombosis in the literature. SerpinB5, ERRa, and IGFBP-5 measurements in COVID-19 patients were mainly lower than healthy controls and exhibited a positive correlation with PTT; however, similar to PCMT1, none of the correlated proteins have been linked to thrombosis previously. Hemoglobin was negatively correlated with fibronectin in COVID-19 patients, with all patients having fibronectin levels lower than healthy controls. MammaglobinA, a secreted glycosylated proteins involved in cell signalling and the immune response, differentiated COVID-19 patients who received high-flow nasal cannula oxygen therapy as an intervention [54, 55]. Lastly, ProSAAS, a neuroendocrine hormone, was lower in those patients with pre-existing hypertension [56].

Serpins are a family of protease inhibitors that use conformational changes to inhibit target enzymes [57]. Four of the 28 proteins that changed in COVID-19 were Serpins (A1, D1, A4, and A12), and all were downregulated. In line with a previous study, SerpinA1 was downregulated in our COVID-19 cohort [58]. SerpinA1 is proposed to limit SARS-CoV-2 cell entry via inhibition of cell surface transmembrane protease 2 (TMPRSS2) function, a critical step in the required processing of the SARS-CoV-2 spike protein [59]. In addition, SerpinA1 was associated with decreased COVID-19 severity [60, 61], and suggested as a potential COVID-19 treatment. Indeed, COVID-19 patients with moderate to severe acute respiratory distress syndrome improved in a phase 2 randomized control trial after SerpinA1 intervention [62]. Administration of SerpinA1 is also suggested as a therapy for alpha-1-antitrypsin deficiency (AATD), in which there is an increased risk of emphysema, obstructive lung disease, and liver disease [61–68]; however, it is unclear if AATD mutations are associated with COVID-19 severity [61, 69, 70]. SerpinD1, a thrombosis inhibitor [71], competes with the SARS-CoV-2 spike protein to bind heparin, resulting in increased thrombosis risk [72]. The regulation of SerpinD1 in COVID-19 is controversial, as a study has shown that SerpinD1 was higher in moderate and severe cases [73]. SerpinA4, also known as kallistatin, exerts multiple effects on inflammation, angiogenesis, and tumor growth. A single nucleotide polymorphism in the SerpinA4 gene was linked to acute kidney injury in COVID-19 patients [74]. Down-regulation of SerpinA4 was noted in COVID-19 non-survivors, indicating a persistent pro-inflammatory signature [75]. SerpinA12 is an adipokine that has been linked to the development of insulin resistance, obesity, and inflammation [76]. In COVID-19, the downregulation of SerpinA12 may heighten inflammation via the kallikrein–kinin system [77].

NLP analysis processed expert-curated expression information from the UniProt Knowledgebase to identify organ- and cell-specific proteins. Of the 28 proteins, 14 (50%) had organ system expression information, with most proteins linked to expression in the digestive and nervous systems. NLP cell-type analysis results were inconclusive, as only eight proteins had cell-type expression information.

Gastrointestinal system complications are prevalent in COVID-19 patients, including diarrhea, nausea/vomiting, and abdominal pain [9, 78, 79]. Fen1, involved in critical DNA synthesis and repair mechanisms, was overexpressed in our COVID-19 cohort. Fen1 is reported to be involved in hepatocellular and gastrointestinal cancers [80, 81], and a novel antiviral strategy that utilizes FEN1 to decrease SARS-CoV-2 cellular functions has been proposed [82]. The expression of both CrkL and fibronectin was decreased in our COVID-19 cohort. The former, which is associated with gastrointestinal cancers, has been suggested as a potential COVID-19 drug target [83–85]. The latter is a widely expressed extracellular matrix protein associated with liver regeneration, fibrogenesis, and intestinal inflammation [86–88].

Nervous system symptoms in COVID-19 patients are prevalent, with COVID-19 severity being associated with increased neurological complications [89–91]. Our NLP analysis identified proteins, mainly down-regulated, from our COVID-19 cohort that are linked to the nervous system. SHANK1, downregulated in COVID-19 patients, facilitates protein-protein interactions in excitatory synapses [92], and its downregulation may hinder neuronal communication [93]. Our COVID-19 patients had decreased expression of PCMT1, a carboxyl methyltransferase. PCMT1 downregulation is linked to neurodegenerative diseases and may increase ß-amyloid production [94, 95]. PARK7 is decreased in our COVID-19 patients and may not effectively perform its protective role against neurotoxicity and neuronal viability [96–98]. PARK7 performs various cellular functions, including acting as a chaperone, interacting with transcription factors, and being involved in anti-oxidative properties under oxidative stress conditions [99–101]. PARK7 is a critical protein involved in the gut-brain axis and related to altered gut microbiomes [102, 103]. Nucleobinding 1 (NUCB1) is widely expressed in brain neurons and stabilizes amyloid protofibrils before they mature and become harmful in neurodegenerative diseases [104, 105]; however, its downregulation in our COVID-19 patients suggests decreased neurological protective mechanisms. Presenilin2 is a crucial protein in neurodegenerative disease and was decreased in our COVID-19 patients. Presenilin2 is responsible for the cleaving enzymatic action required to form amyloid plaques and also forms Ca^2+^ leak channels that support the calcium hypothesis of AD [106–109]. Similar to Presenillin2, ProSAAS, an amyloid anti-aggregant in Alzheimer’s disease, is decreased in our COVID-19 patients [110]. ProSAAS is a neuroendocrine chaperone protein with protective effects against neurodegeneration, such that increased endocrine and neurological cell stressors are associated with elevated expression [111, 112]. Galanin was downregulated in our COVID-19 patients and operates on the neuroendocrine axis with various functions throughout the central and peripheral nervous and endocrine systems [113]. Fyn, elevated in our COVID-19 cohort, has a harmful role in neurological diseases and may be a potential target for neurodegenerative disease due to its ß-amyloid signalling and tau interactions [114–116].

NLP analysis also identified the endocrine system as potentially impacted due to differential protein expression. COVID-19 patients with hypertension had significantly lower expression of ProSAAS, which may be related to ProSAAS peptides involved in salt sensitivity [117]. Diabetes diagnosis and insulin sensitivity have been linked to COVID-19 severity and mortality [118–120], and downregulated ERRa in our COVID-19 cohort is linked to insulin resistance, diabetes, and obesity [121–124]. ERRa regulates glycolysis and lipid metabolism in multiple organs, along with steroidogenesis in the adrenal cortex [125–127]. Similar to our cohort, lower IGFBP-5 expression was previously observed in COVID-19 patients [128], and IGFBPs are linked to diabetes and metabolic disorders [129–133]. SerpinA12 was down-regulated in our COVID-19 patients and is associated with diabetes and obesity due to its insulin-sensitizing effects [134–138]. The downregulated NUCB1 in our COVID-19 patients suggested a harmful effect related to type 2 diabetes as it performs amyloid stabilization in human islet cells to prevent fibrils in the pancreas that impact type 2 diabetes [104, 139, 140]. The decreased PARK7 in COVID-19 patients could also be connected to a metabolic imbalance. PARK7 protects pancreatic beta-cells from oxidative stress conditions, and its deficiency is associated with decreased inflammatory and adipogenesis responses [141–143] and type 2 diabetes [144, 145]. Lastly, Presenilin 2 is expressed in endocrine cells, but there is insufficient data on its role and association with diabetes [146, 147].

COVID-19 is linked with various cardiovascular changes, including vascular transformation, thrombosis, and angiogenesis [148–153]. NLP analysis revealed proteins expressed in the cardiovascular system. GATA-4 is involved in cardiac remodelling, differentiation, and signalling by acting as a cardiogenic transcription factor [154–156]. GATA-4 was reduced in our COVID-19 patients, indicating that subsequent remodelling pathways may be impaired. IGFBP-5 expression was reduced in COVID-19 patients [128], and it is an inhibitor of angiogenesis and vascular smooth muscle cell proliferation [157–159]. PF4V1, decreased in our COVID-19 patients, is an angiogenesis inhibitor and may also regulate inflammation and thrombosis [160–163]. SerpinA4 (Kallistatin) was lower in our COVID-19 patients [164], and it protects against vascular oxidative stress and inflammation as well as inhibiting angiogenesis [165–167]. Thus, the decreased expression of IGFBP-5, PF4V1, and SeprinA4 in COVID-19 may be cardioprotective, perhaps via suppression of angiogenesis and vascular transformation. EphB4, also associated with angiogenesis, was downregulated in our COVID-19 patients [168–171].

The novelties of this study include the proteins identified, the immune microarray platform utilized, and several of the analytic techniques. Previous proteomics studies have also identified molecular models that differentiate COVID-19 patients from non-COVID-19 sepsis controls and healthy control participants [172–175]. While these studies identify a number of important molecules, they did not evaluate their effectiveness in a single combined model, which decreases the likelihood of cross-identity concerns with other diseases. The novel proteins identified in our study may be attributed to our use of an immune microarray platform, while other studies utilized mass spectrometry or proximity extension assays [172–177]. Pathway analysis was used in previous studies to help understand COVID-19 pathophysiology [174, 176, 177]; however, our approach utilized NLP to identify organ and cell expression patterns.

In this study, we identified a novel 28-protein signature and an optimal 9-protein signature that accurately classifies COVID-19 patients from non-COVID-19 sepsis controls and healthy control subjects; however, our study has several limitations. First, the number of subjects in each comparison group was limited to 15, which impacted the choice of analytic. Conservative methods were used to avoid common overfitting or non-generalizable results. Conventional statistics consisted of only non-parametric methods with strict multiple comparison correction. Machine learning classification utilized cross-validation with conservative parameters and without any hyperparameter tuning. Also, protein model building and testing consisted of separate data subsets to reduce overfitting. Second, not all identified proteins had UniProt Knowledgebase-curated expression information, leaving the potential for unrecognized patterns in organ and cell system expression. Similarly, there is a possibility for missed organ/cell identification with NLP; however, preprocessing of expression information was carefully done, and NER used a state-of-the-art biomedical model. Third, static protein measurements must be interpreted with caution as they do not always correlate with functional changes. As one example, Serpins undergo a conformational change to elicit biological effects and therefore require further functional analyses. Lastly, we only compared the COVID-19 proteome signatures to other cohorts, but there may be cross-identity concerns with other illnesses. The use of multiple proteins would reduce this latter limitation. Although our exploratory study had these minor constraints, the data provided insight into the pathophysiological changes in COVID-19 patients.

## Conclusion

Our understanding of COVID-19 pathophysiology, especially in critically ill patients, is incomplete due to its multi-system complications. We identified 28 proteins that accurately differentiate COVID-19 ICU patients from non-COVID-19 sepsis ICU controls and healthy control subjects. The leading proteins are expressed in multiple organ systems and are associated with various diseases and pathophysiological functions, including diabetes, neurodegeneration, metabolic processes, and vascular transformation. The results of our proteomic exploratory study offer insightful information about COVID-19 and might aid in the development of future treatments.

### Electronic supplementary material

Below is the link to the electronic supplementary material.

Supplementary Material 1

## Data Availability

The datasets generated and/or analysed during the current study are available from the corresponding author on reasonable request.

## Abbreviations

AUC: Area-under-the-curve ROC
COVID-19: Coronavirus Disease 2019
FiO_2_: Fractional Inspired Oxygen
ICU: Intensive Care Unit
INR: International Normalized Ratio
MODS: Multiple Organ Dysfunction Score
NER: Named Entity Recognition
NLP: Natural Language Processing
PaO_2_: Partial Pressure of Oxygen
PTT: Partial Thromboplastin Time
RFE: Recursive Feature Elimination
ROC: Receiver Operating Characteristic Curves
SARS: Severe Acute Respiratory Syndrome
SARS-CoV-2: Severe Acute Respiratory Syndrome Coronavirus 2
SOFA: Sequential Organ Failure Assessment Score
t-SNE: T-distributed Stochastic Nearest Neighbor Embedding algorithm
