# Supplementary material for: A reduced proteomic signature in critically ill Covid-19 patients determined with plasma antibody micro-array and machine learning
Source: Clin Proteomics. 2024 May 17;21:33. doi: 10.1186/s12014-024-09488-3 (PMC11100131; doi:10.1186/s12014-024-09488-3)
Supplement: Supplementary file 1 [file 12014_2024_9488_MOESM1_ESM.docx]

**Supplemental Table 1: ROC Area-Under-the-Curve (AUC) Analysis and Feature Importance of the 28 Proteins for Healthy vs COVID-19 ICU**

| **Protein** | **Healthy Controls** **RFI** | **ICU COVID-19** **RFI** | **ROC Logistic AUC** | **F1** | **Specificity** | **Sensitivity** | **Feature Importance %** |
| --- | --- | --- | --- | --- | --- | --- | --- |
| Nucleobindin1/NUCB1 | 2139.6 (2035.6-2526.9) | 1715.6 (1500.7-1977.1) | 0.90 | 0.77 | 0.81 | 0.76 | 1.85 |
| Fibronectin | 45978.1 (41953.6-50954.6) | 29829.7 (25145.5-32101.9) | 0.98 | 0.21 | 0.98 | 0.22 | 8.63 |
| SerpinB5 | 31377.6 (22662.9-34870.9) | 18502.8 (14779.7-21861.6) | 0.83 | 0.15 | 0.95 | 0.16 | 1.16 |
| HSPA8 | 5332.2 (4701.7-6263.1) | 2951.1 (2420.5-4056.6) | 0.87 | 0.75 | 0.80 | 0.76 | 2.12 |
| ERRa | 3357.6 (3191.4-3812.2) | 1301.7 (1107.9-1780.1) | 0.99 | 0.94 | 0.94 | 0.95 | 11.00 |
| SerpinA12 | 39283.3 (29516.8-42026.8) | 21207.7 (17283.3-26846.4) | 0.86 | 0.15 | 0.95 | 0.15 | 1.66 |
| Fyn | 2347.3 (2083.6-2509.2) | 3838.5 (3208.9-4700.4) | 0.96 | 0.92 | 0.96 | 0.89 | 10.81 |
| GATA-4 | 1334.7 (1135.0-1454.9) | 606.8 (485.2-660.6) | 0.97 | 0.94 | 0.95 | 0.93 | 10.27 |
| MammaglobinA | 4569.8 (4269.2-5600.4) | 2944.7 (2434.6-3529.7) | 0.91 | 0.83 | 0.88 | 0.83 | 4.99 |
| SerpinD1 | 2622.1 (2282.0-2897.9) | 766.5 (623.2-1239.1) | 0.98 | 0.94 | 0.94 | 0.93 | 10.57 |
| Presenilin2 | 1007.6 (904.5-1145.2) | 629.2 (571.3-727.1) | 0.94 | 0.85 | 0.85 | 0.85 | 3.64 |
| SerpinA4 | 46385.1 (39766.8-48938.7) | 19742.5 (18351.1-24913.0) | 0.96 | 0.27 | 0.96 | 0.27 | 5.59 |
| PARK7 | 1008.3 (938.2-1224.0) | 390.1 (235.5-486.8) | 0.94 | 0.87 | 0.90 | 0.86 | 3.76 |
| IGFBP-5 | 1327.5 (1216.5-1438.3) | 808.3 (681.8-977.9) | 0.82 | 0.79 | 0.82 | 0.78 | 1.09 |
| HPR | 4088.8 (3254.0-5490.0) | 2559.9 (1905.0-2956.7) | 0.91 | 0.80 | 0.77 | 0.83 | 2.07 |
| EphB4 | 2039.2 (1753.0-2486.6) | 781.8 (661.0-1212.6) | 0.95 | 0.82 | 0.83 | 0.83 | 3.32 |
| Fen1 | 1959.4 (1586.4-2183.9) | 3452.5 (3196.2-3699.9) | 0.96 | 0.89 | 0.92 | 0.87 | 6.72 |
| SHANK1 | 2106.9 (1993.2-2526.0) | 1646.0 (1420.8-1958.0) | 0.81 | 0.68 | 0.75 | 0.69 | 0.39 |
| CrkL | 10605.7 (6730.2-15438.9) | 3775.9 (3093.4-7097.2) | 0.81 | 0.49 | 0.78 | 0.52 | 0.77 |
| Azurocidin | 736.7 (649.8-914.3) | 1249.0 (1041.2-1458.9) | 0.91 | 0.83 | 0.84 | 0.83 | 4.33 |
| PCMT1 | 2732.7 (1904.8-2965.8) | 1699.3 (1395.0-1943.7) | 0.84 | 0.74 | 0.67 | 0.80 | 1.14 |
| SerpinA1 | 56368.0 (49094.9-65906.2) | 38358.2 (35020.4-47527.1) | 0.79 | 0.07 | 0.97 | 0.07 | 0.67 |
| Proteasome26SS5 | 2571.5 (2123.1-3126.2) | 1827.8 (1709.8-1996.8) | 0.83 | 0.78 | 0.73 | 0.83 | 0.65 |
| PF4V1 | 2995.3 (2579.3-3466.0) | 2172.4 (2067.1-2461.0) | 0.86 | 0.73 | 0.74 | 0.77 | 1.00 |
| Galanin | 61227.5 (52182.3-70312.9) | 46296.2 (34583.3-55448.2) | 0.72 | 0.06 | 0.95 | 0.07 | 0.36 |
| ProSAAS | 79695.5 (55365.2-90619.9) | 43001.7 (36282.0-79197.1) | 0.66 | 0.10 | 0.89 | 0.13 | 0.62 |
| VimentinB | 1348.7 (1236.9-1570.0) | 1082.3 (757.1-1418.7) | 0.76 | 0.63 | 0.70 | 0.62 | 0.41 |
| NET1 | 6309.6 (5222.1-7691.0) | 7573.7 (6916.4-8694.5) | 0.69 | 0.64 | 0.17 | 0.90 | 0.41 |

**Supplemental Table 2: ROC Area-Under-the-Curve (AUC) Analysis and Feature Importance of the 28 Proteins for non-COVID-19 ICU vs COVID-19 ICU**

| **Protein** | **ICU non-COVID-19** **RFI** | **ICU COVID-19** **RFI** | **ROC Logistic AUC** | **F1** | **Specificity** | **Sensitivity** | **Feature Importance %** |
| --- | --- | --- | --- | --- | --- | --- | --- |
| Nucleobindin1/NUCB1 | 2767.9 (2349.5-3960.4) | 1715.6 (1500.7-1977.1) | 0.97 | 0.89 | 0.87 | 0.91 | 5.73 |
| Fibronectin | 48802.8 (38226.8-60780.7) | 29829.7 (25145.5-32101.9) | 0.91 | 0.18 | 0.95 | 0.19 | 1.96 |
| SerpinB5 | 36270.3 (28011.4-42295.1) | 18502.8 (14779.7-21861.6) | 0.96 | 0.24 | 0.96 | 0.25 | 3.78 |
| HSPA8 | 5589.2 (4712.2-6968.6) | 2951.1 (2420.5-4056.6) | 0.94 | 0.85 | 0.86 | 0.86 | 4.62 |
| ERRa | 3900.5 (3328.8-4948.5) | 1301.7 (1107.9-1780.1) | 0.98 | 0.94 | 0.93 | 0.95 | 10.05 |
| SerpinA12 | 55427.7 (45914.9-65692.0) | 21207.7 (17283.3-26846.4) | 0.95 | 0.18 | 0.96 | 0.19 | 3.48 |
| Fyn | 1734.6 (1677.4-2289.4) | 3838.5 (3208.9-4700.4) | 0.93 | 0.84 | 0.85 | 0.84 | 2.48 |
| GATA-4 | 1070.0 (877.2-1186.5) | 606.8 (485.2-660.6) | 0.89 | 0.86 | 0.83 | 0.89 | 2.48 |
| MammaglobinA | 4873.9 (3900.4-5373.9) | 2944.7 (2434.6-3529.7) | 0.87 | 0.74 | 0.81 | 0.76 | 0.96 |
| SerpinD1 | 2533.9 (1626.6-3197.0) | 766.5 (623.2-1239.1) | 0.95 | 0.84 | 0.80 | 0.88 | 1.89 |
| Presenilin2 | 968.2 (884.8-1061.5) | 629.2 (571.3-727.1) | 0.83 | 0.82 | 0.84 | 0.82 | 0.57 |
| SerpinA4 | 43038.2 (36277.4-51919.7) | 19742.5 (18351.1-24913.0) | 0.94 | 0.16 | 0.97 | 0.16 | 3.46 |
| PARK7 | 809.6 (627.4-1145.1) | 390.1 (235.5-486.8) | 0.87 | 0.78 | 0.73 | 0.81 | 1.07 |
| IGFBP-5 | 1437.6 (1257.0-1797.8) | 808.3 (681.8-977.9) | 0.88 | 0.79 | 0.78 | 0.80 | 2.51 |
| HPR | 4883.1 (4162.4-6666.2) | 2559.9 (1905.0-2956.7) | 0.95 | 0.91 | 0.87 | 0.95 | 4.39 |
| EphB4 | 2281.4 (1431.5-2747.9) | 781.8 (661.0-1212.6) | 0.89 | 0.76 | 0.73 | 0.78 | 0.66 |
| Fen1 | 1701.8 (1165.3-2907.6) | 3452.5 (3196.2-3699.9) | 0.67 | 0.62 | 0.56 | 0.74 | 0.46 |
| SHANK1 | 3637.3 (2935.6-4592.3) | 1646.0 (1420.8-1958.0) | 0.94 | 0.87 | 0.83 | 0.91 | 2.28 |
| CrkL | 13671.4 (8277.4-23294.7) | 3775.9 (3093.4-7097.2) | 0.92 | 0.64 | 0.81 | 0.67 | 2.76 |
| Azurocidin | 786.3 (458.5-980.9) | 1249.0 (1041.2-1458.9) | 0.84 | 0.75 | 0.75 | 0.77 | 0.30 |
| PCMT1 | 2860.9 (2242.7-4468.6) | 1699.3 (1395.0-1943.7) | 0.95 | 0.86 | 0.83 | 0.88 | 2.78 |
| SerpinA1 | 92996.3 (72774.3-110845.8) | 38358.2 (35020.4-47527.1) | 0.99 | 0.09 | 0.99 | 0.09 | 8.85 |
| Proteasome26SS5 | 2930.7 (2679.6-3663.4) | 1827.8 (1709.8-1996.8) | 0.90 | 0.86 | 0.81 | 0.90 | 1.54 |
| PF4V1 | 4117.6 (3632.9-4974.6) | 2172.4 (2067.1-2461.0) | 0.93 | 0.90 | 0.89 | 0.92 | 5.73 |
| Galanin | 96782.7 (88396.2-103526.7) | 46296.2 (34583.3-55448.2) | 1.00 | 0.09 | 1.00 | 0.09 | 12.32 |
| ProSAAS | 124482.4 (107774.4-140386.0) | 43001.7 (36282.0-79197.1) | 0.92 | 0.08 | 0.98 | 0.08 | 1.37 |
| VimentinB | 2424.8 (1881.2-3601.1) | 1082.3 (757.1-1418.7) | 0.93 | 0.90 | 0.85 | 0.94 | 4.92 |
| NET1 | 3231.5 (2128.9-4140.6) | 7573.7 (6916.4-8694.5) | 0.96 | 0.91 | 0.91 | 0.92 | 6.61 |

**Supplemental Table 3. Function of Reduced 28 Proteins**

| **Biomarker** | **Function** |
| --- | --- |
| Nucleobindin1/NUCB1 | Nucleobindin-1 (NUCB1) is a calcium-binding protein found in the Golgi and expressed in neurons in all brain regions as well as endocrine tissues (1, 2). NUCB1 is also linked with ECM degradation and invasive cell migration in macrophages (3). It may also be involved in diabetes mechanisms as per current animal model studies (1, 4). |
| Fibronectin | Fibronectin is a large glycoprotein in the ECM that is involved in ECM structure as well as cell adhesion, signalling, and growth (5). Plasma fibronectin circulates in large concentrations and is deposited in ECM while cellular fibronectin is secreted locally (6). |
| SerpinB5 | Mammary Serine Protease Inhibitor (SerpinB5, Maspin) is classified as part of the serpin family of serine proteases inhibitors, however, it does not exhibit the ability due to no conformational transition characteristic (7). There is contradictory evidence on SerpinB5 as a tumor suppressor and critical role in embryogenesis (8, 9). SerpinB5 is associated with mammary gland morphogenesis and cornea cell adhesion (10, 11). |
| HSPA8 | Heat shock protein family A (HSP70) member 8 (HSPA8) is a constitutively expressed protein with various cellular functions. With other chaperones, HSPA8 enables correct protein folding of a wide range of nascent polypeptides. HSPA8 also plays a role in clathrin-mediated endocytosis, cellular protein degradation and protein import (12). HSPA8 is also upregulated when stresses there are stressed in the immune system enabling cell signalling and acting as an antigen (13). |
| ERRa | Estrogen-related receptor alpha (ERRa) is a nuclear receptor and is important in regulating cellular metabolism by acting as a transcription factor (14). It regulates genes involved in glycolysis including glucose transporters, PDK isoenzyme 4, lactate dehydrogenase as well as lipid metabolism and oxidative phosphorylation mechanisms (15). It is linked to various cancers and metabolic diseases including type 2 diabetes and cardiac (16-19). |
| SerpinA12 | Visceral adipose tissue-derived serpin (SerpinA12, Vaspin) is a serine protease secreted by visceral adipose tissue and is identified to be involved in insulin-sensitizing and glucose tolerance in diabetes and obesity (20, 21). A possible mechanism is via inhibition of Kallikrein-related peptidase 7 (KLK7) which cleaves insulin (22). |
| Fyn | Tyrosine-protein kinase Fyn (Fyn) is a member of the Src family with various functions and is also labelled as a proto-oncogene (23). Fyn is involved in T-cell functions including the T-cell antigen receptor (TCR) signal transduction (24). Also, Fyn has been associated with abnormal phosphorylation of tau protein as well as other mechanisms involved with Alzheimer’s Disease (25, 26). |
| GATA-4 | GATA-4 is a zinc-finger transcription factor part of the GATA family. GATA-4 is involved in cardiac function and development with animal models showing important functions in cardiac muscle structure and differentiation as well as cardiogenesis (27-29). GATA-4 is also associated with testicular and ovarian development (30, 31). |
| MammaglobinA | Secretoglobin family 2A member 2 (MammaglobinA) is a secreted protein that is primarily expressed in the breast epithelial tissue (32). While the exact function is unknown, it is a key biomarker in breast cancer diagnosis (33, 34). |
| SerpinD1 | Heparin cofactor 2 (SerpinD1), part of the serpin family, is a thrombin inhibitor with cofactors heparin and dermatan sulfate (35). Its function inhibits thrombosis and has also been associated as a protective factor for atherosclerosis (36, 37) |
| Presenilin2 | Presenilin2 (PSEN2) is in the endoplasmic reticulum membrane and is linked to Alzheimer’s disease (AD), specifically familial AD (FAD) (38). PSEN2 functions as a gamma-secretase and cleaves beta-amyloid precursor protein (APP) to form the amyloid beta-peptide responsible for amyloid plaques in AD (39). PSEN2 is also responsible for forming Ca^2+ ^leak channels in ER membrane linked to the calcium hypothesis of AD (40-42). |
| SerpinA4 | Kallistatin (SeprinA4) belongs to the serine protease family and is synthesized in the liver and secreted. Kallistatin, via the active site, is an inhibitor of tissue kallikrein, a protease necessary in the production of vasoactive peptide kinin (43). Alternatively, through its heparin-binding site, it is also associated with blocking various signalling pathways associated with decreased angiogenesis, inflammation, and tumor growth (44-46). |
| PARK7 | Parkinson’s disease protein 7 (PARK7) aka protein deglycase DJ-1 is involved in oxidative stress conditions and functions as a neuroprotective protein (47). The protein controls protein synthesis by interacting with transcription factors and RNA (48). |
| IGFBP-5 | Insulin-like growth factor (IGF) binding protein 5 (IGFBP-5) is part of the IGF signalling pathway. Due to its ability to regulate IGF signalling, IGFBP-5 can regulate cell proliferation, growth and migration (49). It is also suspected to be involved in the development of atherosclerotic plaques and regulator of vascular smooth muscle cells (50, 51). |
| HPR | Haptoglobin-related protein (HPR) is associated with apolipoprotein L-I (apoL-I)-containing high-density lipoprotein (HDL) particles and binds to hemoglobin with high affinity (52, 53). HDL-containing HPR has been linked to antimicrobial molecules with a role in innate immunity (54). |
| EphB4 | Ephrin type-B receptor 4 (EphB4) is a tyrosine kinase receptor that performs bidirectional cross-talk signalling via binding to other cells’ ephrin-B ligands (55). EphB4 is involved in vascular transformation including vascular maturation, angiogenesis, and endothelial cell sorting (56-58). |
| Fen1 | Flap endonuclease 1 (Fen1) is a critical enzyme that removes the 5’ overhangs from single-stranded DNA in DNA synthesis and repair (59). Fen1 mutations have been proposed to cause genomic instability and are linked primarily to cancer but also inflammation and autoimmunity (60-62). |
| SHANK1 | SH3 and multiple ankyrin repeat domains protein 1 (SHANK1) is a postsynaptic density (PSD) protein found in excitatory synapses and facilitates protein-protein interactions. It functions as a scaffold protein for the organization and stability of cytoskeletal and signalling complexes in the neuron (63). Downregulation of SHANK1 is associated with decreased number of active synapses (64). |
| CrkL | Crk-like protein (CRKL) is part of essential signal transduction pathways interacting with various activation sources (65). As such, it is involved in cell growth, proliferation and migration as well as cell and pathogen death (66). |
| Azurocidin | Azurocidin also known as heparin-binding protein (HBP) is released by neutrophils for their gram-negative antibacterial activity (67). While it primarily attracts monocytes and macrophages in bacterial infections, it also alters vascular permeability (67, 68). |
| PCMT1 | Protein-L-isoaspartate (D-aspartate) O-methyltransferase (PCMT1) enzyme is a type II class protein carboxyl methyltransferase that does DNA repair via conversion of incorrect D-aspartyl and L-isoaspartyl to normal L-aspartyl. Its function has also been suggested to be necessary to maintain MAP kinase signalling (69). |
| SerpinA1 | Alpha-1-antitrypsin (SerpinA1, A1AT) is part of the Serpins family which inhibits serine proteases. SeprinA1 is secreted into the plasma and primarily inhibits elastase along with other proteases. Alpha-1-antitrypsin deficiency (AATD) due to inherited genetic variations results in an increased risk for emphysema and obstructive lung disease, especially in smokers (70-72). SerpinA1 is also associated with liver disease as mutants cannot be secreted from hepatocyte ER (73-75). |
| Proteasome26SS5 | 26S proteasome non-ATPase regulatory subunit 5 (Proteasome26SS5, PSMD5) is a chaperone for the assembly of the 26S proteasome (76). The 26S proteasome is a part of the ubiquitin-proteasome system responsible for protein degradation as per cell processes and protein hemostasis (77). |
| PF4V1 | Platelet factor 4 variant (PF4V1, CXCL4L1) is a chemokine that is released by platelets and involved in inhibiting angiogenesis and tumor growth (78 , 79). It also can chemoattract T cells, natural killer cells and immature dendritic cells and is implicated in predicting coronary artery disease patient outcomes (80, 81). |
| Galanin | Galanin is a neuropeptide that signals via G protein-coupled receptors in the central and peripheral nervous system and the endocrine system. It has a variety of functions including, but not limited to, altering neurotransmitter release and cognitive functions as well as cellular signalling (82, 83). |
| ProSAAS | ProSAAS is a neuroendocrine secretory chaperone protein that inhibits prohormone convertase 1 (PC1) which activates precursor proteins (84, 85). It is also noted to function as an amyloid anti-aggregant in Alzheimer’s disease (86). |
| VimentinB | VimentinB is a class three intermediate filament structural protein that is part of the cytoskeleton and functions to maintain cell shape and support organelles. It is noted to be an epithelial-mesenchymal transition and fibroblast marker as well as regulate signalling pathways involved in cell migration and ECM remodelling (87). It has also been implicated in various diseases including viral infection (88, 89). |
| NET1 | Neuroepithelial cell-transforming gene 1 protein (NET1), a Rho guanine nucleotide exchange factor, activates Rho proteins, possibly after DNA damage signalling (90). NET1 may be involved in cell morphology via transforming growth factor beta (TGF-B) signalling pathways (91, 92). |

**Supplemental Table 4: Organ System Expression Keyword Categories and NLP Associated Proteins**

| **Organ System** | **Proteins** | **Keywords** |
| --- | --- | --- |
| Cardiovascular | EphB4, Fibronectin, Nucleobindin 1/NUCB1, PARK7, Presenilin 2 | abdominal aorta, adventitial connective, aorta, aorta extracellular, aortic, aortic intima, aortic smooth muscle, arteries, arterioles, artery, ascending aorta, atherosclerotic vessels, atrial, atrium, blood vessel walls, blood vessels, brain microvascular, capillaries, cardiac, cardiac atria, cardiac muscle, cardiovascular, cardiovascular system, cerebral vessels, coronary arteries, coronary artery, dermal blood vessels, endocardium, endothelial, endothelial venules, endothelium, fetal cardiac, fetal heart, heart, heart muscle, lateral ventricle, lateral wall, outflow tract, parenchymal vessels, pericardium, periosteum, skin blood vessels, small vessels, stromal vascular, subendothelial layer, suprarenal artery, system vessels, thoracic aorta, umbilical cord vascular smooth muscle, vascular, vascular capillary network, vascular endothelium, vascular smooth muscle, vascular structure, vascular-rich organs, vasculature, veins, ventricle, villous endothelium, visceral smooth muscle |
| Digestive | EphB4, HPR, IGFBP-5, Nucleobindin 1/NUCB1, PARK7, Presenilin 2, ProSAAS, Serpin A4, Serpin D1 | adult pancreas, adult stomach, appendix, bile ducts, bowel, brush border, buccal mucosa, colon, colon epithelium, colon mucosa, colon mucosal lining, colon parasympathetic ganglia, colonic crypts, colonic epithelium, colonic lining, colonic mucosa, colorectal, crypt base, crypts, cystic duct, deodenum, descending colon, digestive, digestive system epithelium, duodenum, duodenum mucosal crypts, esophageal epithelium, esophageal submucosa, esophagus, exocrine pancreatic ducts, fetal colon, fetal liver, fundic epithelium, gall bladder, gallbladder, gastric, gastric epithelium, gastric mucosa, gastrointestinal, gastrointestinal tract, gingival mucosa, gut, hepatic endothelia, ileum, intestinal, intestinal crypts, intestinal epithelia, intestinal epithelium, intestinal mucosa, intestinal tract, intestine, intestines, islets, jejunum, jejunum brush, lamina propria, langerhans, large intestine, lingual mucosa, liver, liver hepatocytes, mouth, mucous acini, muscularis mucosa, normal stomach, omental, oral cavity, oral epithelia, oral epithelium, oral mucosa, palatal epithelia, palatal mucosa, palatal shelf, palate, pancreas, pancreas kidney, pancreatic, pancreatic acini, pancreatic fluid, pancreatic islets, parotid gland, parotid salivary gland intralobular ducts, rectum, salivary, salivary gland, salivary glands, serous gland ducts, sinusoidal epithelium, small intestine, stomach, sublingual gland, submandibular, submandibular gland, submaxillary glands, tongue, tongue mesenchyme, tooth, tract epithelia, transverse colon, villi, villous stroma |
| Endocrine | EphB4, Nucleobindin 1/NUCB1, PARK7, Presenilin 2, Serpin A12 | adipose, adrenal, adrenal cortex, adrenal gland, arcuate nucleus, brown adipose, ducts, endocrine, endocrine glands, fat, fetal adrenal, fetal hypothalamus, gland, glands, glandular duct, glandular epithelium, hypophysis, hypothalamus, lacrimal gland, parathyroid, parathyroid gland, parathyroid glands, pituitary, pituitary gland, placenta, placenta vascular endothelium, placental, placental stem villi vessels, placental villi, secretory epithelia, steroidogenic glands, subcutaneous adipose, submaxillary gland, submucosal gland, submucosal glands, suprarenal capsule, term placenta, thyroid, thyroid gland, thyroid glands, ventral thalamus, visceral adipose, visceral fat, white adipose, white fat |
| Integumentary | PARK7 | ) hair, adult skin suprabasal layers, anagen, anagen follicles, basal cell layer, basal epithelia, basal epithelium, basal layer, body wall, club hair, cornified epidermis, cuticle, dermal papilla, dermis, eccrine apparatus, eccrine sweat, epidermal, epidermal sweat gland ducts, epidermal-dermal junction, epidermis, epidermis stratum corneum, exocrine, eyebrow, eyelid, fetal skin, fetal skin epidermis, follicles, follicular, follicular fluid, hair, hair bulb, hair follicle, hair follicles, hair shaft, infundibular outer root sheath, inner root sheath, inner root sheaths, irs cuticle, lesional psoriatic skin, lower stratum corneum, nail, nail bed epithelium, nail bed mesenchyme, nail matrix, outer root sheath, palmar skin, palmoplantar epidermis, primary follicles, psoriatic epidermis, rete ridges, scalp, scalp hair follicles, sebaceous gland, sebaceous glands, skin, skin adnexal structures, skin epidermis, spinosum, stratum corneum, stratum granulosum, stratum spinosum, suprabasal layers, sweat, sweat ducts, sweat gland, sweat gland ducts, sweat glands |
| Musculoskeletal | EphB4, Nucleobindin 1/NUCB1, PARK7, Presenilin 2 | appendicular skeleton, arrector pili muscle, articular, articular cartilage, articular cartilages, bone, bone matrix, bone-forming sites, bone-forming surfaces, bones, calvaria, carpal bones, cartilage, cartilaginous, cartilaginous cores, cortical plate, deep zone cartilage, dentin, enamel organ, epiphysis, femur, fetal cartilage, fetal skeletal muscle, fetal skeletal muscles, ganglia, gastrocnemious muscle, hypertrophic cartilage, inner periosteal region, invertebral disk, joint capsule, joint cartilage, ligament, ligaments, long bone, long bones, metaphyseal bone, muscle, muscle fibers, oa lesions, osseous, osteoarthritic synovium, perichondrium, pulp, rheumatoid synovial, rib bone, rib cartilage, skeletal, skeletal muscle, skeletal muscles, smooth muscle, smooth muscle layers, smooth muscles, striated muscle, striated muscles, subchondral bone, superficial zone, synaptic fibers, synovial, synovial fluid, synovial fluids, synovium, tarsal bones, tendon, tibia, trabecular bone, vertebrae, visceral smooth muscle |
| Nervous | Fibronectin, Fyn, IGFBP-5, Nucleobindin 1/NUCB1, PARK7, Presenilin 2, ProSAAS, SHANK1 | -brain barrier, adrenal medulla, adult brain, adult cns, adult nervous central system, amygdala, anterior horn, anterior perisylvian cortex-opercular gyri, astrocyte-like structures, auerbach plexus, basal ganglia, blood-brain, brain, brain areas, brain cortex, brain stem, brain structures, brains, caudate, caudate nucleus, central, central nervous, central nervous system, cerebellar nuclei, cerebellum, cerebral cortex, cerebral spinal, cerebro-spinal fluid, choroid plexus, ciliary body, ciliary border, ciliary nonpigmented epithelium, cns, cochlea, cochlear nerve, conjunctiva, conjunctival epithelia, cornea, corneal, corneal epithelium, corneal stromal layer, corpus callosum, corpus luteum, corpus region, cortex, corti, cranial ganglia, dentate gyrus, diencephalon, dorsal root ganglia, dura, embryonic retina, eye, eye anterior segment, eye lens, fetal brain, fetal cerebellum, forebrain, frontal cortex, frontal lobe, frontotemporal lobes, ganglion cell layer, germinal neuroepithelium, hindbrain, hippocampus, inner nuclear layer, inner plexiform layer, insula, iris, lacrimal, lens, lens capsule, lens vesicle, limbic system, lumbar, medulla, medulla oblongata, medulla region, medullary area, meibomian glands, midbrain, midbrain structures, motor cortices, neocortex, neocortical regions, nerve, nerve bundles, nerve fiber layer, nervous system, neural, neuron, neuronal, notochord, nucleus accumbens, occipital lobe, occipital pole, ocular, olfactory bulb, olfactory epithelium, olfactory tubercles, ophthalmic nerve, optic cup, optic nerve, outer nuclear layer, outer plexiform layers, parahippocampal cortex, parahippocampal cortices, paraolfactory gyri, paraventricular nucleus, parietal lobe, peripheral nerve, peripheral nervous system, peripheral nervous systems, peripherical nerve, periventricular, periventricular part, photoreceptor outer, pns neuroectoderm, pons, posterior perisylvian, prefrontal cortex, putamen, retina, retina pigment epithelium, retinal pericytes, retinal pigment epithelia, retinal pigment epithelium, rolandic area, rostral segment, sciatic nerve, spinal chord, spinal cord, spinal cordon, spiral ganglions, striate, striated tracts, striatum, subcortical nuclei, subependymal layer, substantia nigra, subthalamic nuclei, subthalamic nucleus, superior colliculus, sustantia nigra, sympathetic, synaptic fibers, taste buds, telencephalon, temporal cortex, temporal gyrus, temporal lobe, thalamus, ventrolateral part, vertebrae, vestibular system |
| Reproductive | EphB4, Mammaglobin A, PARK7 | adult testis, bartholin's, breast, breast cyst, cerebrum, cervix, chorioamniotic mesenchyma, chorion, chorionic villi, decidua, decidua basalis, ectocervical epithelium, endocervical glands, endocervix, endometrial, endometrial fluid, endometrial stroma, endometrium, endometrium basalis, epididymal glands, epididymis, exocervix, fallopian tube, fallopian tubes, female reproductive, fetal gonads, fetal ovary, fetal testis, foreskin, genital, germinal zone, gingival crevicular fluid, graaf follicle fluids, mammary, mammary epithelial cell surfaces, mammary gland, mammary glands, myometrium, neoplastic prostate, nipple aspirate, nipple epidermis, oocytes, ovarian, ovaries, ovary, penis, placenta liver, prostate, prostate gland, reproductive, reproductive system, seminal vesicle, seminal vesicles, testes, testis, testis kidney, transitional epithelium, umbilical chord, umbilical cord, uterine endometrium, uterine fluid, uterine glandular epithelium, uterus, vagina, vaginal epithelium, yolk sac |
| Respiratory | EphB4, Nucleobindin 1/NUCB1 | adenoids, adult lung, aerobic, airway, airway epithelium, airway sputum, airways, alveolar epithelium, alveolar walls, bronchial, bronchial epithelial, bronchiolar, bronchus, distal parenchyma, fetal lung, larynx, lung, nasal cavity, nasal epithelium, nasal mucosa, nasal septal epithelium, nasopharyngeal regions, nasopharynx, pharynx, pseudostratified epithelia, pulmonary, pulmonary airways, pulmonary alveoli, respiratory epithelium, respiratory tract, ribs, sinonasal, trachea, tracheobronchial, upper airways |
| Urinary | EphB4, IGFBP-5, Nucleobindin 1/NUCB1, PARK7, Presenilin 2 | adult bladder, bladder, bladder epithelia, bladder urothelium, bowman's capsules, collecting tubules, convoluted tubule, convoluted tubule lumen, fetal kidney, fetal kidneys, glomerular slit diaphragm, glomeruli, glomerulus, kidney, kidney distal, kidney proximal, kidneys, muscularis propria, nephron, non tumor kidney, pancreas kidney, proximal tubules, renal proximal tubule, testis kidney, tubular epithelium, ureter, urinary bladder, urinary tracts, urogenital, urothelium, vas deferens, vasa rectae |
| Lymphatic | -- | adenoid, adult thymus, afferent lymphatics, bone marrow, fetal bone marrow, fetal spleen, fetal thymus, fetal tonsils, germinal center, hassal's corpuscles, hematopoietic, immune system, interstitial fluid, lymph, lymph node, lymph node-containing, lymph nodes, lymph nodes subcapsular, lymphatic vessels, lymphatics, lymphoid, lymphoid organs, medullary sinuses, mesenteric lymph nodes, peripheral lymph nodes, peyer patches, red pulp, spleen, thymus, thymus epithelium, tonsil, tonsillar lymphatic sinuses, tonsils |

**Supplemental Table 5: Cell Type Expression Keyword Categories and NLP Associated Proteins.**

| **Cell Type** | **Proteins** | **Keywords** |
| --- | --- | --- |
| Myeloid | EphB4 | myeloid, myeloid lines, myeloid lineage, myeloid progenitor, myeloma line u266b1, myeloma line u266r |
| Neuron | EphB4 | brain neurons, cerebellar purkinje, cortical neurons, dorsal root ganglia neurons, dorsal root ganglion, gabaergic neurons, gray matter neurons, hippocampus pyramidal neurons, lines, neural, neural crest, neural stem, neuroendocrine, neuron, neuronal, neurons, purkinje neurons, pyramidal neurons, schwann, spinal cord neurons, striatal neurons |
| Skin | EphB4 | basal keratinocytes, cornified, epidermal basal layer keratinocytes, epidermal keratinocytes, keratinocyte, keratinocytes, melanocyte, melanocytes, skin keratinocytes, suprabasal keratinocytes |
| Fibroblast | Fibronectin | cerebral pericytes, dermal fibroblasts, fetal fibroblasts, fibroblast, fibroblast lines, fibroblast-like synoviocytes, fibroblastic, fibroblasts, foreskin fibroblasts, myofibroblasts, pulmonary fibroblasts, skin fibroblasts, stromal fibroblast, synovial, synovial fibroblasts, synovial fluid |
| Liver | Fibronectin | crypt, hepatic, hepatic parenchymal, hepatic stellate, hepatocytes, hepatoma, liver hepatocytes |
| Epithelial | Fibronectin, HPR, Serpin B5 | a-549, alveolar epithelial, alveolar type 2, antral epithelial, bronchial, bronchial epithelial, choroid plexus epithelial, columnar epithelial, corneal epithelial, embryonic epithelial, epithelial, epithelial  lines, eue, gastrointestinal epithelial, hela, helas3, hep-g2, intestinal epithelial, lung alveolar type 2, lung epithelial, mammary epithelial, mcf-7, mesothelial, myoepithelial, myoepithelium, nasal, nasal epithelial, nonciliated, paneth, parietal epithelial, pharyngeal epithelial, prostate gland epithelial, retinal pigment epithelial, secretory epithelial, thymic, thymic epithelial, tubular epithelial, vascular epithelial |
| Lymphocyte | Fyn | b, b-, b- lines, b-lymphocytes, cytotoxic t-lymphocytes, gamma-delta t-, germinal centers, helper t-, hsb, jurkat t- leukemia, lymphoblasts, lymphocyte, lymphocytes, lymphocytic lineage, lymphoid, mature b, memory th17, molt-4, naive t, natural killer, natural killer (nk), neoplastic b- and t- lines, nk, nkt, normal germinal center (gc) b-, pbl, peripheral blood lymphocytes, peripheral blood t-, peripheral blood t-lymphocytes, peripheral t-, sup-t1, t, t lymphocytes, t-, t- clones, t- lines, t-helper, t-helper 2, t-lymphoblasts, t-lymphocytes, th0, th1, treg, yt |
| Glial | PARK7 | astrocytes, astrocytoma, glia, glial, glioma, glioma lines, glioma tissue, liver astrocytes, microglia, microglial, oligodendrocytes, perivascular astrocytes |
| Reproductive | PARK7 | cumulus, extravillous trophoblast, gonocytes, granulosa, interstitial leydig, leydig, myometrial, oocytes, ovarian granulosa, ovary, postnatal leydig, prostate, sertoli, spermatocytes, spermatogonia, spermatozoa, syncytiotrophoblasts, trophoblast, trophoblasts, x |
| Leukocytes Nyd | Serpin A1 | blood mononuclear, bone marrow, bone marrow-derived mesenchymal stem, flattened bone-lining, immune, interstitial leukocytes, leukocyte, leukocytes, mast, mononuclear, normal mast, peripheral blood leukocyte, peripheral blood leukocytes, peripheral blood mononuclear, peripheral blood mononuclear leukocytes, peripheral leukocyte, peripheral leukocytes, peripheral mononuclear, phagocytic, plasma, polymorphonuclear leukocytes, promyelocytes, promyelocytic, submucosal leukocytes, thymocytes, urothelial, white blood |
| Cancer | Vimentin B | acute myelogenous leukemia), breast cancer  lines, cancer, cancer  lines, carcinoma, carcinoma  lines, choriocarcinoma cancer  lines, colon adenocarcinoma  lines, erythroleukemia, fibrosarcoma, gastric, glioblastomas, hel, hepatoular carcinoma, hl-60, k-562, k-562 erythroleukemia, kidney tumor, leiomyomal, leukemia  lines, leukemia u-937, leukemia u-937  line, leukemic, lung carcinoma  lines, lymphoma, lymphoma  lines, malignant, mammary carcinoma  lines, mcf-7 breast carcinoma, melanoma, melanoma  lines, metastasizing melanoma  lines, myelogenous leukemia  line kg-1, myelogenous leukemic  lines, myeloid leukemia  lines, nb4, neuro-epithelioma, neuroblastoma, non invasive breast carcinoma  lines, nurse-like, pancreatic cancer  lines, pc-3, prostate cancer  lines, retinoblastoma  lines, several cancer  lines, sk-ov-3 (ovary adenocarcinoma), sw480, sw480 colon carcinoma, tumor, tumor  lines, tumor-derived  lines, tumors  lines, u-937 histiocytic lymphoma  lines |
| Adipocyte |  | adipocytes |
| Chondrocyte |  | articular chondrocytes, chondrocytes |
| Dendritic |  | dc, dendritic, follicular dendritic, immature dendritic, monocyte-derived, monocyte-derived dendritic, myeloid dendritic, peripheral blood plasmacytoid dendritic, thymic dendritic |
| Dental |  | ameloblast, cementoblast, odontoblast |
| Endocrine |  | enterocytes, enteroendocrine, enteroendocrine l, ileal absorptive enterocytes |
| Endothelial |  | angioblasts, aortic endothelial, arterial endothelial, artery endothelial, blood brain barrier endothelial, cervical epithelium, umbilical vein endothelial, endiothelial, endothelial, human umbilical vein endothelial, huvecs, lymph vessel endothelial, placenta, umbilical vein endothelial, vascular endothelial |
| Erythrocyte |  | erythrocytes, erythroid, fetal erythrocytes |
| Eye |  |  |
| Granulocyte |  | basophils, eosinophils, granulocyte, granulocytes, inflammatory, neutrophil, neutrophil lineage, neutrophils, peripheral blood granulocytes |
| Hematopoietic |  | hematopoetic, hematopoietic, hematopoietic lines, hematopoietic stem |
| Kidney |  | bowman's capsule, embryonal kidney, glomerular epithelium, hsc, interstitial, kidney distal tubular, podocyte, podocytes, proximal tubule, renal, renal lines, renal proximal tubular, tubular |
| Macrophage |  | a, alveolar macrophages, bone marrow macrophages, cortical macrophages, epidermoid, hofbauer, kg-1, kupffer, langerhans, langerhans', liver kupffer, liver kuppfer, lung alveolar macrophages, m1 macrophages, macrophage, macrophage progenitor, macrophage-like, macrophages, meningeal macrophages, perivascular macrophages, red pulp macrophages, tissue macrophages |
| Monocytes |  | monocyte, monocytes, monocytic, myelomonocytic lineage, peripheral blood monocytes, promonocytic, thp-1 |
| Mucous |  | bronchial goblet, goblet, nasal goblet secretory, upper gastric mucosal |
| Muscle |  | arterial smooth muscle, artery smooth muscle, cardiomyocytes, muscle, myoblasts, myocytes, myotubes, perivascular, placental vascular smooth muscle, pulmonary artery smooth muscle, skeletal muscle, smooth, smooth muscle, vascular, vascular smooth muscle, vascular wall, vsmc |
| Non-Hematopoietic |  | non-hematopoietic |
| Osteoblast |  | bone osteoblasts, osteoblast, osteoblasts, osteoclasts, osteocytes, osteogenic, primary ossification center-associated |
| Other |  | epidermal basal, inner ear hair, intimal, non-neuronal, nonmuscle, villous |
| Other Blood |  | blood, blood lines, peripheral blood, red blood, reticulocytes |
| Pancreatic |  | pancreatic lines, pancreatic acinar, pancreatic islet |
| Platelet |  | eosinophil platelets, megakaryocytes, megakaryocytic, platelet, platelets, thrombocytes |
| Secretory |  | chief, chromaffin, fundic, gastric parietal, secretory, weibel-palade bodies |
| Spleen |  | fetal spleen |
| Stem |  | cambial, cml) stem, embryonic stem, esc, mesenchymal, mesenchymal stem |
| Stromal |  | endometrial stromal, stromal, stromal type |

**SUPPLEMENTAL FIGURE LEGENDS:**

**Supplemental Figure 1: Flow diagram of data processing and machine learning methodology. A)** Flow diagram depicting the plasma sample processing with background subtraction, normalization, and the creation of a dataset ready for analysis. **B)** Flow diagram depicting the training (70%) and testing (30%) data split, feature selection process with Boruta and Recursive Feature Elimination, and assessment of classification ability. **C)** Flow diagram depicting the datasets used in t-SNE unsupervised clustering.

**Supplemental Figure 2: Box plots of normalized protein expression data.** Following background subtraction and normalization, the median protein expression values from each patient cohort are similar.

**Supplemental Figure 3: Unsupervised Clustering comparing healthy controls and ICU non-COVID-19 patients to ICU COVID-19 patients on Day 1 (all 2,000 proteins) A)** Healthy controls compared to ICU COVID-19 Day 1 measurements plotted in two dimensions, following t-SNE dimensionality reduction of all measured proteins. The plot shows some cluster separation in ICU COVID-19 patients, but with six overlapping patients. **B)** ICU non-COVID-19 compared to ICU COVID-19 Day 1 measurements plotted in two dimensions, following t-SNE dimensionality reduction of all measured proteins. The plot shows a distinct cluster separation of ICU COVID-19 patients from ICU non-COVID-19 patients. 

**Supplemental Figure 4: Relative Fluorescence Intensity comparison of ICU COVID-19 patients for reduced 28 proteins. A)** Intensity plot showing the fold change of each COVID-19 patient on Day 1 normalized to the healthy control median, separately for all 28 proteins. There are 4 proteins (Fyn, Fen1, Azurocidin, and Net1) that have the majority of the COVID-19 patients with an elevated fold change, while all other 24 proteins show decreased expression relative to healthy controls. **B)** Intensity plot showing fold change of each COVID-19 patient on Day 1 normalized to ICU non-COVID-19 patients’ median, separately for all 28 proteins. There are 4 proteins (Fyn, Fen1, Azurocidin, and Net1) that have the majority of the COVID-19 patients with an elevated fold change, while all other 24 proteins show decreased expression relative to ICU non-COVID-19 patients.

**Supplemental Figure 5: Recursive Feature Selection of 28 Protein Results after 10000 Runs** The plot showcases the number of Recursive Feature Selection runs each protein made it into the top 10. The 5000-run threshold (50%) is shown with an orange dashed line.

**Supplemental Figure 6:** **Frequency of protein expression in cell types.** A bar plot demonstrates the percentage of proteins that are expressed in specific cell types as determined by Natural Language Processing. There was a total of 8 proteins out of the 28 proteins (29%) with UniProt cell type expression information. Only those cell types with at least one protein expression are shown for visualization clarity.

**Supplemental Figure 1:** 

**Supplemental Figure 2:** 

**Supplemental Figure 3:** 

**Supplemental Figure 4:** 

**Supplemental Figure 5:** 

**Supplemental Figure 6:**

**Supplemental References:**

1.	Williams P, Tulke S, Ilegems E, Berggren P-O, Broberger C. (2014) Expression of nucleobindin 1 (NUCB1) in pancreatic islets and other endocrine tissues. *Cell and Tissue Research* **358:** 331-342.

2.	Tulke S*, et al.* (2016) Nucleobindin 1 (NUCB1) is a Golgi-resident marker of neurons. *Neuroscience* **314:** 179-188.

3.	Pacheco-Fernandez N*, et al.* (2020) Nucleobindin-1 regulates ECM degradation by promoting intra-Golgi trafficking of MMPs. *Journal of Cell Biology* **219**.

4.	Bonito-Oliva A, Barbash S, Sakmar TP, Graham WV. (2017) Nucleobindin 1 binds to multiple types of pre-fibrillar amyloid and inhibits fibrillization. *Scientific reports* **7:** 1-12.

5.	Dalton CJ, Lemmon CA. (2021) Fibronectin: Molecular Structure, Fibrillar Structure and Mechanochemical Signaling. *Cells* **10**.

6.	Maurer LM, Ma W, Mosher DF. (2016) Dynamic structure of plasma fibronectin. *Critical Reviews in Biochemistry and Molecular Biology* **51:** 213-227.

7.	Pemberton PA*, et al.* (1995) The Tumor Suppressor Maspin Does Not Undergo the Stressed to Relaxed Transition or Inhibit Trypsin-like Serine Proteases.: EVIDENCE THAT MASPIN IS NOT A PROTEASE INHIBITORY SERPIN (∗). *Journal of Biological Chemistry* **270:** 15832-15837.

8.	Teoh SSY*, et al.* (2014) Maspin is not required for embryonic development or tumour suppression. *Nature Communications* **5:** 3164.

9.	Zou Z*, et al.* (1994) Maspin, a Serpin with Tumor-Suppressing Activity in Human Mammary Epithelial Cells. *Science* **263:** 526-529.

10.	Ngamkitidechakul C, Burke JM, O’Brien WJ, Twining SS. (2001) Maspin: synthesis by human cornea and regulation of in vitro stromal cell adhesion to extracellular matrix. *Investigative ophthalmology & visual science* **42:** 3135-3141.

11.	Bodenstine TM*, et al.* (2012) Maspin: molecular mechanisms and therapeutic implications. *Cancer and Metastasis Reviews* **31:** 529-551.

12.	Stricher F, Macri C, Ruff M, Muller S. (2013) HSPA8/HSC70 chaperone protein. *Autophagy* **9:** 1937-1954.

13.	Bonam SR, Ruff M, Muller S. (2019) HSPA8/HSC70 in Immune Disorders: A Molecular Rheostat that Adjusts Chaperone-Mediated Autophagy Substrates. In:  *Cells.*

14.	Tripathi M, Yen PM, Singh BK. (2020) Estrogen-Related Receptor Alpha: An Under-Appreciated Potential Target for the Treatment of Metabolic Diseases. *International Journal of Molecular Sciences* **21:** 1645.

15.	Audet-walsh É, Giguére V. (2015) The multiple universes of estrogen-related receptor α and γ in metabolic control and related diseases. *Acta Pharmacologica Sinica* **36:** 51-61.

16.	Handschin C, Mootha VK. (2005) Estrogen-related receptor α (ERRα): A novel target in type 2 diabetes. *Drug Discovery Today: Therapeutic Strategies* **2:** 151-156.

17.	Wang T*, et al.* (2015) Estrogen-Related Receptor α (ERRα) and ERRγ Are Essential Coordinators of Cardiac Metabolism and Function. *Molecular and Cellular Biology* **35:** 1281-1298.

18.	May FEB. (2014) Novel drugs that target the estrogen-related receptor alpha: their therapeutic potential in breast cancer. *Cancer Management and Research* **6:** 225-252.

19.	Larsen LH*, et al.* (2007) Genetic analysis of the estrogen-related receptor α and studies of association with obesity and type 2 diabetes. *International Journal of Obesity* **31:** 365-370.

20.	Hida K*, et al.* (2005) Visceral adipose tissue-derived serine protease inhibitor: a unique insulin-sensitizing adipocytokine in obesity. *Proc Natl Acad Sci U S A* **102:** 10610-10615.

21.	Yang W, Li Y, Tian T, Wang L. (2017) Serum Vaspin Concentration in Elderly Type 2 Diabetes Mellitus Patients with Differing Body Mass Index: A Cross-Sectional Study. *Biomed Res Int* **2017:** 4875026.

22.	Heiker JT*, et al.* (2013) Vaspin inhibits kallikrein 7 by serpin mechanism. *Cell Mol Life Sci* **70:** 2569-2583.

23.	Resh MD. (1998) Fyn, a Src family tyrosine kinase. *The International Journal of Biochemistry & Cell Biology* **30:** 1159-1162.

24.	Palacios EH, Weiss A. (2004) Function of the Src-family kinases, Lck and Fyn, in T-cell development and activation. *Oncogene* **23:** 7990-8000.

25.	Lee G*, et al.* (2004) Phosphorylation of Tau by Fyn: Implications for Alzheimer's Disease. *The Journal of Neuroscience* **24:** 2304-2312.

26.	Larson M*, et al.* (2012) The Complex PrP<sup>c</sup>-Fyn Couples Human Oligomeric Aβ with Pathological Tau Changes in Alzheimer's Disease. *The Journal of Neuroscience* **32:** 16857-16871.

27.	Molkentin JD, Kalvakolanu DV, Markham BE. (1994) Transcription Factor GATA-4 Regulates Cardiac Muscle-Specific Expression of the α-Myosin Heavy-Chain Gene. *Molecular and Cellular Biology* **14:** 4947-4957.

28.	Grépin C, Robitaille L, Antakly T, Nemer M. (1995) Inhibition of Transcription Factor GATA-4 Expression Blocks In Vitro Cardiac Muscle Differentiation. *Molecular and Cellular Biology* **15:** 4095-4102.

29.	Ang YS*, et al.* (2016) Disease Model of GATA4 Mutation Reveals Transcription Factor Cooperativity in Human Cardiogenesis. *Cell* **167:** 1734-1749.e1722.

30.	Ketola I*, et al.* (2000) Expression of Transcription Factor GATA-4 during Human Testicular Development and Disease1. *The Journal of Clinical Endocrinology & Metabolism* **85:** 3925-3931.

31.	Heikinheimo M*, et al.* (1997) Expression and Hormonal Regulation of Transcription Factors GATA-4 and GATA-6 in the Mouse Ovary*. *Endocrinology* **138:** 3505-3514.

32.	Al Joudi FS. (2014) Human mammaglobin in breast cancer: a brief review of its clinical utility. *Indian J Med Res* **139:** 675-685.

33.	Zehentner BK, Carter D. (2004) Mammaglobin: a candidate diagnostic marker for breast cancer. *Clinical Biochemistry* **37:** 249-257.

34.	Han J-H*, et al.* (2003) Mammaglobin Expression in Lymph Nodes Is an Important Marker of Metastatic Breast Carcinoma. *Archives of Pathology & Laboratory Medicine* **127:** 1330-1334.

35.	Tollefsen D, Pestka CA, Monafo W. (1983) Activation of heparin cofactor II by dermatan sulfate. *Journal of Biological Chemistry* **258:** 6713-6716.

36.	He L, Vicente CP, Westrick RJ, Eitzman DT, Tollefsen DM. (2002) Heparin cofactor II inhibits arterial thrombosis after endothelial injury. *The Journal of Clinical Investigation* **109:** 213-219.

37.	Aihara K, Azuma H, Akaike M, Sata M, Matsumoto T. (2009) Heparin cofactor II as a novel vascular protective factor against atherosclerosis. *J Atheroscler Thromb* **16:** 523-531.

38.	Annaert WG*, et al.* (1999) Presenilin 1 controls γ-secretase processing of amyloid precursor protein in pre-Golgi compartments of hippocampal neurons. *The Journal of cell biology* **147:** 277-294.

39.	Kimberly WT, Xia W, Rahmati T, Wolfe MS, Selkoe DJ. (2000) The transmembrane aspartates in presenilin 1 and 2 are obligatory for gamma-secretase activity and amyloid beta-protein generation. *J Biol Chem* **275:** 3173-3178.

40.	Tu H*, et al.* (2006) Presenilins form ER Ca2+ leak channels, a function disrupted by familial Alzheimer's disease-linked mutations. *Cell* **126:** 981-993.

41.	Berridge MJ. (2010) Calcium hypothesis of Alzheimer’s disease. *Pflügers Archiv - European Journal of Physiology* **459:** 441-449.

42.	Zampese E*, et al.* (2011) Presenilin 2 modulates endoplasmic reticulum (ER)-mitochondria interactions and Ca2+ cross-talk. *Proc Natl Acad Sci U S A* **108:** 2777-2782.

43.	Chao J, Schmaier A, Chen L-M, Yang Z, Chao L. (1996) Kallistatin, a novel human tissue kallikrein inhibitor: Levels in body fluids, blood cells, and tissues in health and disease. *Journal of Laboratory and Clinical Medicine* **127:** 612-620.

44.	Chao J, Bledsoe G, Chao L. (2016) Protective Role of Kallistatin in Vascular and Organ Injury. *Hypertension* **68:** 533-541.

45.	Miao RQ, Agata J, Chao L, Chao J. (2002) Kallistatin is a new inhibitor of angiogenesis and tumor growth. *Blood* **100:** 3245-3252.

46.	Lin W-C*, et al.* (2015) Kallistatin protects against sepsis-related acute lung injury via inhibiting inflammation and apoptosis. *Scientific Reports* **5:** 12463.

47.	Junn E, Jang WH, Zhao X, Jeong BS, Mouradian MM. (2009) Mitochondrial localization of DJ-1 leads to enhanced neuroprotection. *Journal of Neuroscience Research* **87:** 123-129.

48.	Huang M, Chen S. (2021) DJ-1 in neurodegenerative diseases: Pathogenesis and clinical application. *Progress in Neurobiology* **204:** 102114.

49.	Duan C, Allard JB. (2020) Insulin-Like Growth Factor Binding Protein-5 in Physiology and Disease. *Frontiers in Endocrinology* **11**.

50.	Xu Q*, et al.* (2004) Evidence That IGF Binding Protein-5 Functions as a Ligand-Independent Transcriptional Regulator in Vascular Smooth Muscle Cells. *Circulation Research* **94:** e46-e54.

51.	Frystyk J, Ledet T, Møller N, Flyvbjerg A, Ørskov H. (2002) Cardiovascular Disease and Insulin-Like Growth Factor I. *Circulation* **106:** 893-895.

52.	Skytthe MK*, et al.* (2022) Haptoglobin-related protein in human plasma correlates to haptoglobin concentrations and phenotypes. *Scand J Clin Lab Invest* **82:** 461-466.

53.	Nielsen MJ*, et al.* (2006) Haptoglobin-related protein is a high-affinity hemoglobin-binding plasma protein. *Blood* **108:** 2846-2849.

54.	Shiflett AM, Bishop JR, Pahwa A, Hajduk SL. (2005) Human high density lipoproteins are platforms for the assembly of multi-component innate immune complexes. *J Biol Chem* **280:** 32578-32585.

55.	Chrencik JE*, et al.* (2006) Structural and Biophysical Characterization of the EphB4&#xb7;EphrinB2 Protein-Protein Interaction and Receptor Specificity *. *Journal of Biological Chemistry* **281:** 28185-28192.

56.	Füller T, Korff T, Kilian A, Dandekar G, Augustin HG. (2003) Forward EphB4 signaling in endothelial cells controls cellular repulsion and segregation from ephrinB2 positive cells. *J Cell Sci* **116:** 2461-2470.

57.	Du E, Li X, He S, Li X, He S. (2020) The critical role of the interplays of EphrinB2/EphB4 and VEGF in the induction of angiogenesis. *Molecular Biology Reports* **47:** 4681-4690.

58.	Groppa E*, et al.* (2018) EphrinB2/EphB4 signaling regulates non-sprouting angiogenesis by VEGF. *EMBO reports* **19:** e45054.

59.	Tsutakawa Susan E*, et al.* (2011) Human Flap Endonuclease Structures, DNA Double-Base Flipping, and a Unified Understanding of the FEN1 Superfamily. *Cell* **145:** 198-211.

60.	Zheng L*, et al.* (2007) Fen1 mutations result in autoimmunity, chronic inflammation and cancers. *Nature Medicine* **13:** 812-819.

61.	Mason PA, Cox LS. (2012) The role of DNA exonucleases in protecting genome stability and their impact on ageing. *AGE* **34:** 1317-1340.

62.	Yang M*, et al.* (2009) Functional FEN1 polymorphisms are associated with DNA damage levels and lung cancer risk. *Human Mutation* **30:** 1320-1328.

63.	Sheng M, Kim E. (2000) The Shank family of scaffold proteins. *J Cell Sci* **113 ( Pt 11):** 1851-1856.

64.	Shi R*, et al.* (2017) Shank Proteins Differentially Regulate Synaptic Transmission. *eNeuro* **4**.

65.	Birge RB, Kalodimos C, Inagaki F, Tanaka S. (2009) Crk and CrkL adaptor proteins: networks for physiological and pathological signaling. *Cell Communication and Signaling* **7:** 13.

66.	Feller SM. (2001) Crk family adaptors-signalling complex formation and biological roles. *Oncogene* **20:** 6348-6371.

67.	Soehnlein O, Lindbom L. (2009) Neutrophil-derived azurocidin alarms the immune system. *Journal of Leukocyte Biology* **85:** 344-351.

68.	Edens HA, Parkos CA. (2003) Neutrophil transendothelial migration and alteration in vascular permeability: focus on neutrophil-derived azurocidin. *Current Opinion in Hematology* **10**.

69.	Furuchi T, Sakurako K, Katane M, Sekine M, Homma H. (2010) The role of protein L-isoaspartyl/D-aspartyl O-methyltransferase (PIMT) in intracellular signal transduction. *Chem Biodivers* **7:** 1337-1348.

70.	Abboud RT, Nelson TN, Jung B, Mattman A. (2011) Alpha1-antitrypsin deficiency: a clinical-genetic overview. *The Application of Clinical Genetics* **4:** 55-65.

71.	Strnad P, McElvaney NG, Lomas DA. (2020) Alpha1-Antitrypsin Deficiency. *New England Journal of Medicine* **382:** 1443-1455.

72.	Yang P*, et al.* (2008) Alpha1-Antitrypsin Deficiency Carriers, Tobacco Smoke, Chronic Obstructive Pulmonary Disease, and Lung Cancer Risk. *Archives of Internal Medicine* **168:** 1097-1103.

73.	Boëlle P-Y, Debray D, Guillot L, Corvol H, on behalf of the French CFMGSI. (2019) SERPINA1 Z allele is associated with cystic fibrosis liver disease. *Genetics in Medicine* **21:** 2151-2155.

74.	Narayanan P, Mistry PK. (2020) Update on Alpha-1 Antitrypsin Deficiency in Liver Disease. *Clin Liver Dis (Hoboken)* **15:** 228-235.

75.	Lomas DA, Li-Evans D, Finch JT, Carrell RW. (1992) The mechanism of Z α1-antitrypsin accumulation in the liver. *Nature* **357:** 605-607.

76.	Roelofs J*, et al.* (2009) Chaperone-mediated pathway of proteasome regulatory particle assembly. *Nature* **459:** 861-865.

77.	Bard JAM*, et al.* (2018) Structure and Function of the 26S Proteasome. *Annual Review of Biochemistry* **87:** 697-724.

78.	Struyf S, Burdick MD, Proost P, Van Damme J, Strieter RM. (2004) Platelets release CXCL4L1, a nonallelic variant of the chemokine platelet factor-4/CXCL4 and potent inhibitor of angiogenesis. *Circ Res* **95:** 855-857.

79.	Vandercappellen J, Van Damme J, Struyf S. (2011) The role of the CXC chemokines platelet factor-4 (CXCL4/PF-4) and its variant (CXCL4L1/PF-4var) in inflammation, angiogenesis and cancer. *Cytokine Growth Factor Rev* **22:** 1-18.

80.	Struyf S*, et al.* (2007) Platelet factor-4 variant chemokine CXCL4L1 inhibits melanoma and lung carcinoma growth and metastasis by preventing angiogenesis. *Cancer research* **67:** 5940-5948.

81.	De Sutter J*, et al.* (2012) PF-4var/CXCL4L1 predicts outcome in stable coronary artery disease patients with preserved left ventricular function. *PLoS One* **7:** e31343.

82.	Vrontakis ME. (2002) Galanin: a biologically active peptide. *Curr Drug Targets CNS Neurol Disord* **1:** 531-541.

83.	Bartfai T, Hökfelt T, Langel U. (1993) Galanin--a neuroendocrine peptide. *Crit Rev Neurobiol* **7:** 229-274.

84.	Fricker LD*, et al.* (2000) Identification and Characterization of proSAAS, a Granin-Like Neuroendocrine Peptide Precursor that Inhibits Prohormone Processing. *The Journal of Neuroscience* **20:** 639-648.

85.	Sayah M, Fortenberry Y, Cameron A, Lindberg I. (2001) Tissue distribution and processing of proSAAS by proprotein convertases. *Journal of Neurochemistry* **76:** 1833-1841.

86.	Hoshino A*, et al.* (2014) A novel function for proSAAS as an amyloid anti-aggregant in Alzheimer's disease. *Journal of Neurochemistry* **128:** 419-430.

87.	Ostrowska-Podhorodecka Z, Ding I, Norouzi M, McCulloch CA. (2022) Impact of Vimentin on Regulation of Cell Signaling and Matrix Remodeling. *Frontiers in Cell and Developmental Biology* **10**.

88.	Zhang Y*, et al.* (2020) The diverse roles and dynamic rearrangement of vimentin during viral infection. *Journal of Cell Science* **134:** jcs250597.

89.	Surolia R, Antony VB. (2022) Pathophysiological Role of Vimentin Intermediate Filaments in Lung Diseases. *Frontiers in Cell and Developmental Biology* **10**.

90.	Dubash AD*, et al.* (2011) The Small GTPase RhoA Localizes to the Nucleus and Is Activated by Net1 and DNA Damage Signals. *PLOS ONE* **6:** e17380.

91.	Shen X*, et al.* (2001) The Activity of Guanine Exchange Factor NET1 Is Essential for Transforming Growth Factor-&#x3b2;-mediated Stress Fiber Formation *. *Journal of Biological Chemistry* **276:** 15362-15368.

92.	Qin H*, et al.* (2005) Characterization of the Biochemical and Transforming Properties of the Neuroepithelial Transforming Protein 1 *. *Journal of Biological Chemistry* **280:** 7603-7613.
